# Common Cell Shape Evolution of Two Nasopharyngeal Pathogens

**DOI:** 10.1371/journal.pgen.1005338

**Published:** 2015-07-10

**Authors:** Frédéric J. Veyrier, Nicolas Biais, Pablo Morales, Nouria Belkacem, Cyril Guilhen, Sylvia Ranjeva, Odile Sismeiro, Gérard Péhau-Arnaudet, Eduardo P. Rocha, Catherine Werts, Muhamed-Kheir Taha, Ivo G. Boneca

**Affiliations:** 1 Institut Pasteur, Infection Bactériennes Invasives, Département Infection et Epidémiologie, Paris, France; 2 Institut Pasteur, Groupe Biologie et Génétique de la Paroi Bactérienne, Département de Microbiologie, Paris, France; 3 INSERM, Groupe Avenir, Paris, France; 4 INRS-Institut Armand-Frappier, Bacterial Symbionts Evolution, Laval, Quebec, Canada; 5 Brooklyn College, Graduate Center City University of New York, Brooklyn, New York, New York, United States of America; 6 Institut Pasteur, Plate-forme de Transcriptome et Epigénome, Département de Génome et Génétique, Paris, France; 7 Institut Pasteur, Plate-forme de Microscopie Ultrastructurale, Département de Biologie Cellulaire et Infection, Paris, France; 8 Institut Pasteur, Microbial Evolutionary Genomics, Département Génome et Génétique, and CNRS, UMR3525, Paris, France; Université Paris Descartes, INSERM U1001, FRANCE

## Abstract

Respiratory infectious diseases are the third cause of worldwide death. The nasopharynx is the portal of entry and the ecological niche of many microorganisms, of which some are pathogenic to humans, such as *Neisseria meningitidis* and *Moraxella catarrhalis*. These microbes possess several surface structures that interact with the actors of the innate immune system. In our attempt to understand the past evolution of these bacteria and their adaption to the nasopharynx, we first studied differences in cell wall structure, one of the strongest immune-modulators. We were able to show that a modification of peptidoglycan (PG) composition (increased proportion of pentapeptides) and a cell shape change from rod to cocci had been selected for along the past evolution of *N*. *meningitidis*. Using genomic comparison across species, we correlated the emergence of the new cell shape (cocci) with the deletion, from the genome of *N*. *meningitidis* ancestor, of only one gene: *yacF*. Moreover, the reconstruction of this genetic deletion in a bacterium harboring the ancestral version of the locus together with the analysis of the PG structure, suggest that this gene is coordinating the transition from cell elongation to cell division. Accompanying the loss of *yacF*, the elongation machinery was also lost by several of the descendants leading to the change in the PG structure observed in *N*. *meningitidis*. Finally, the same evolution was observed for the ancestor of *M*. *catarrhalis*. This suggests a strong selection of these genetic events during the colonization of the nasopharynx. This selection may have been forced by the requirement of evolving permissive interaction with the immune system, the need to reduce the cellular surface exposed to immune attacks without reducing the intracellular storage capacity, or the necessity to better compete for adhesion to target cells.

## Author Summary

The nasopharynx hosts an important microbial community that comprises some well-known pathogens such as *Neisseria meningitidis* and *Moraxella catarrhalis*. In some circumstances, it also represents the portal of entry of systemic infections such as septicemia and meningitis, or infections of the respiratory system, middle ear, eye, central nervous system and joints of humans, caused by *N*. *meningitidis* and *M*. *catarrhalis*, respectively. In this article, we demonstrated that both bacteria underwent a similar cell shape evolution that resulted in a transition from a bacillus to a coccus. This was consequently accompanied by a change, similar for both bacteria, in the structure of the PG, the major bacterial cell shape determinant and also a strongly recognized molecule by the immune system. In our efforts in understanding the evolutionary events that led to the cell shape transition in *N*. *meningitidis*, we identified two genetic deletion events required for the shape transition, i.e. of *yacF* (*zapD*) and the cell elongation machinery. Furthermore, we delineated the importance of YacF (ZapD) in the coordination of the cell elongation and division. Finally, we suggest that this transition was selected to reduce the cell surface sensible to immune attacks and to redistribute surface appendages, such as pili, to acquire new properties of cell adhesion or movement necessary for the proper colonization of the nasopharynx.

## Introduction

Some pathogenic bacteria like *Neisseria meningitidis*, *Streptococcus pneumoniae*, *Haemophilus influenzae*, and *Moraxella catarrhalis* are highly adapted to the ecological niche of the human nasopharynx (NP). This one defines the upper part of the pharynx from the end of nasal cavities (choanoe) to the upper surface of the soft palate. On the lateral parts it communicates with the Eustachian tubes by the pharyngeal ostium whereas the posterior part is composed of the pharyngeal tonsil (adenoids). The aforementioned species are part of the normal human NP microbiome where they generally live in asymptomatic symbiosis. However, some strains can occasionally cause local diseases of the upper-respiratory tract (pharyngitis, laryngitis, bronchitis, sinusitis and otitis) or an invasive infection leading to life threatening diseases, such as pneumonia, septicemia and meningitis.

It is expected that the bacterial adaptation to human mucosa at the NP has occurred through some evolutionary events that allowed immune tolerance of the bacteria by the immune system and/or conferred new properties for bacteria to respond to the novel physical and chemical constraints. In this sense, we hypothesized that modifications of the peptidoglycan (PG), a MAMP (Microbe-Associated Molecular Pattern), may have been selected during NP adaptation. PG is strongly recognized by the host via the specialized receptors NOD1 and NOD2 [1–4]. But, PG is also an essential component of bacterial cell walls that shapes the cell and serves as an exoskeleton conferring resistance to internal turgor pressure [5]. It is composed of polymerized repeats of disaccharide units (N-acetylglucosamine or G and N-acetylmuramic acid or M) cross-linked by short stem peptides. Several reports have already described the selection of different bacterial shapes, varying from rod (bacilli) to spheres (cocci) to helical and spirals (spirochetes), among others, during adaptation to new ecosystems [6–8]. These cell shape changes can be a transition that can happen on the developmental time scale, during a single cell cycle or it can become permanent through the course of bacterial evolution.

Except curvature, cell shape is governed by two mechanisms: cell elongation and division. Numerous proteins allow and orchestrate the spatial and temporal coordination of these mechanisms. Most analyses of Gram-negative cell wall biogenesis have been performed in *Escherichia coli* and are reviewed here [9, 10]. In this model organism, inner membrane-bound penicillin binding proteins (PBPs) are responsible of transglycosylation (polymerization of disaccharide-pentapeptide precursors) and transpeptidation (crosslink of peptide residues). Two bifunctionals PBPs, PBP1A and PBP1B, are central factors of the cell elongation and division machinery, respectively. In addition to PBP1A, the elongation machinery is also composed, among others, of MreB, an actin structural homologue, necessary for a proper localization of the machinery or the monofunctional PBP2 (called PBPX in *Neisseriaceae*) another PBP essential for lateral PG synthesis. The division machinery is, for example, composed of PBP3 (called PBP2 in *Neisseriaceae*) essential for septal/polar PG synthesis.

Due to the high numbers of players and the multiple interconnections, the coordination between cell elongation and division is not yet completely understood, although FtsZ plays a major role. FtsZ is a tubulin-like protein that forms, prior to cell division, a dynamic ring-like structure at the mid-cell that initiates the assembly of the divisome [11]. In this article, we described the evolution from rod-to-cocci of the ancestor of several NP pathogens and show that PG structure evolved during nasopharyngeal adaptation. Furthermore, we show that in the *Neisseriaceae* family, one of the starting points of this evolution event is the deletion of a coordinator of both cell elongation and division called YacF or ZapD. Our data also highlighted changes that accompanied this cell shape transition such as a decreased recognition by the innate immune system, the optimization of the ratio cell surface over the volume or the localization of some surface-exposed structure (e.g. pili).

## Results

### Co-evolution of cell shape and PG structure in the *Neisseriaceae* family

**Fig 1:**
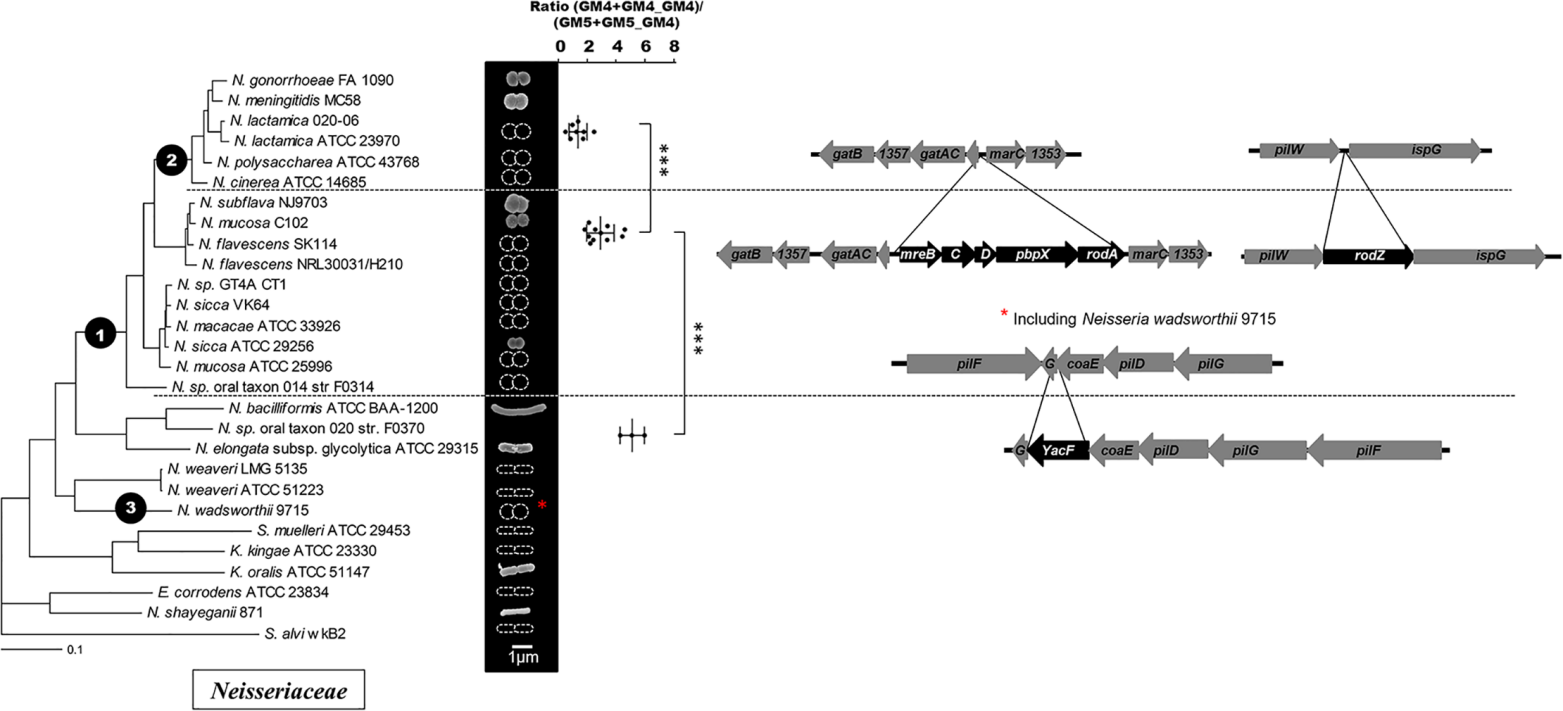
Cell shape evolution among the *Neisseriaceae* family. Phylogeny of the *Neisseriaceae* family based on core genome analysis along with scanning electronic microscopy images of representative species or schematic representation of the cell shape based on the literature. The mean total ratio (GM4+GM4_GM4)/(GM5+GM5_GM4) (with standard deviation), assessed by reverse-phase HPLC, is also presented for the lineages having emerged after the different nodes of evolution (*** p≤0.001; ** p≤0.01; * p≤0.05). Each dote represents an independent isolate (tested for *rodA* presence) from the CNRM collection or from the ATCC collection that have been classified as *N*. *meningitidis* (1), *N*. *lactamica* (2), *N*. *polysaccharea* (1), *N*. *gonorrhoeae* (3), *N*. *cinerea* (1), *N*. *perflava* (1), *N*. *sicca* (3), *N*. *subflava* (2), *N*. *mucosa* (3), *N*. *elongata* (1), *N*. *bacilliformis* (1), *K*. *oralis* (1). Finally, the right part displays the deletions detected at different nodes of evolution by presenting the organization of the different relevant loci in species that diverged before and after the corresponding nodes. Note that the deletion of *yacF* also occurred in *N*. *wadsworthii* suggesting of an independent event at node 3.

Bacteria from the *Neisseriaceae* family have variable cell shape: some are elongated (e.g. *Kingella oralis* or *N*. *elongata*) whereas others present a coccoïd form (e.g. *N*. *meningitidis* and *N*. *gonorrhoeae*). To establish that these differences were linked to the evolution within the family, we correlated the phylogeny (determined using core genome analysis as described in the M&M) with cell shape verified by scanning electronic microscopy for some representative strains that were received at the Centre National de Reference des Méningocoques or with information found in the literature for other well described strains [12] (Fig 1). Our results suggested that *Neisseria* cell shape evolved from rod to coccus at a node of evolution that we have called 1 in the Fig 1. As a note, the coccus *Neisseria wadsworthii* 9715 is related to bacilli *Neisseria* clade with the closest bacterium being the bacillus *Neisseria waeveri*. This phylogeny could be biased by a low quality genomic sequence. Nevertheless it is consistent with previous phylogeny (based on the same genomic sequence) [13]. Therefore, this strain may have undergone an independent coccus cell-shape transition.

As PG is the main determinant of cell shape in bacteria, we determined the muropeptides composition of PG from different *Neisseriaceae* representatives of each lineage. We found a stepwise increase of the proportion of pentapeptide-containing muropeptides (GM5) correlated with a decrease of the proportion of tetrapeptide-containing muropeptides (GM4) along the phylogeny toward *N*. *meningitidis* lineage at node 1 but also at node 2 (Fig 1). This second node corresponded to the divergence of the meningococcal highly related species [14]. Overall, we observed a two-fold reduction of the mean total ratio (GM4+GM4_GM4)/(GM5+GM5_GM4) at node 1 (5.1 vs 2.9) and a further two-fold reduction at node 2 (2.9 vs 1.3).

**Fig 2:**
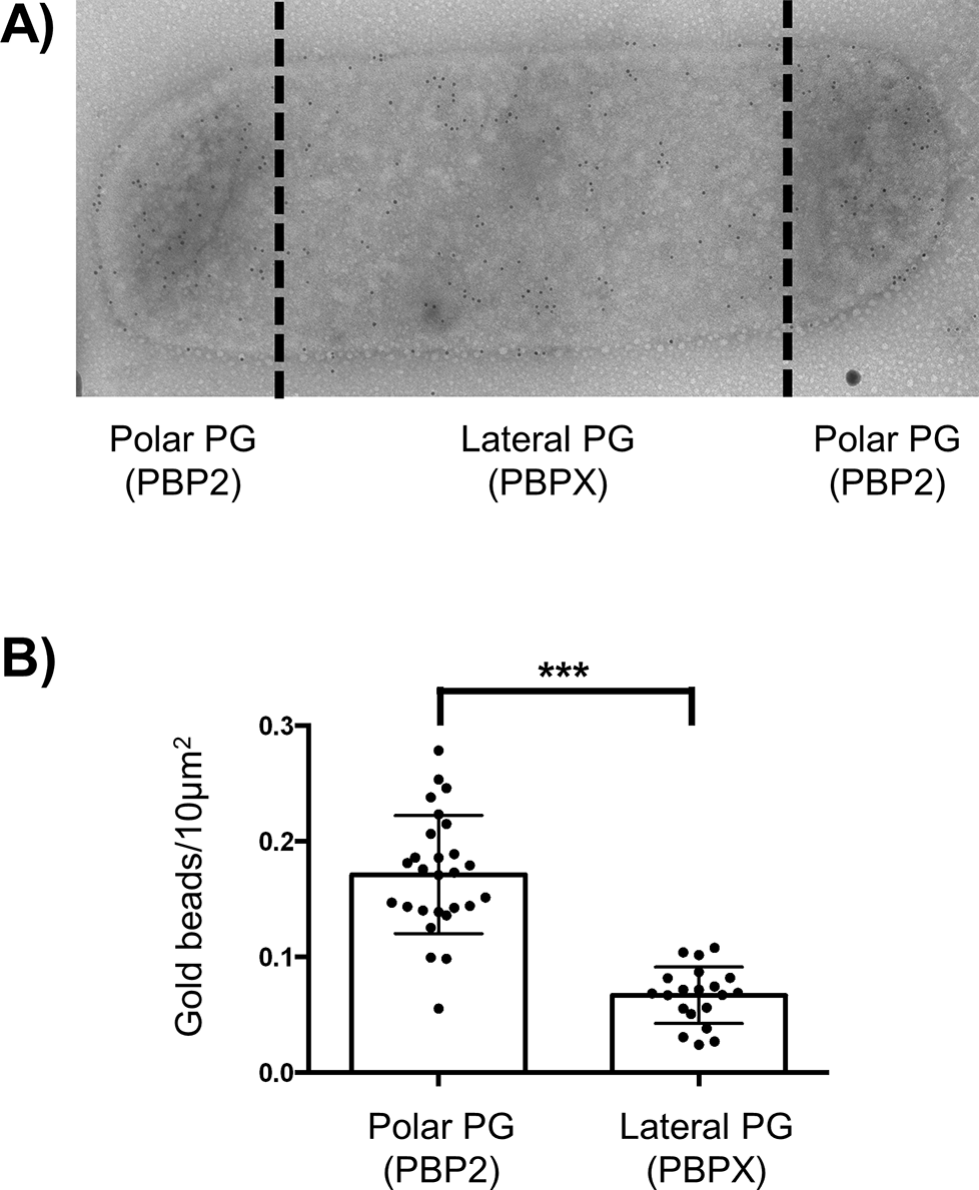
GM5 detection on polar and lateral PG. A) Transmission electronic microscopy image of immuno-gold detection of GM5 using vancomycin labelling of *N*. *bacilliformis* saculli. B) Estimation of the mean numbers, with standard deviation, of gold beads by 10μm^2^ of sacculi surface of both polar/septal and lateral PG measured on around 20 cells (*** p≤0.001).

We hypothesized that this ratio may represent a difference in the structure of the PG septal/polar versus the PG lateral produced during elongation. In other words, the PG septal/polar could be enriched in GM5 containing muropeptides. To test this possibility, we used immune-gold detection of vancomycin binding to the PG saculli of *N*. *bacilliformis* (Fig 2A). The vancomycin has the useful property to specifically bind to GM5 [15]. We measured the number of gold-beads by 10 μm^2^ in both the lateral and polar PG (Fig 2B) and observed an increased occurrence of GM5 in the polar PG. Overall, these results suggest that the change in cell shape led to a global enrichment in GM5 due to a decreased occurrence in the sacculi of lateral PG produced during elongation (less rich in GM5).

### Cell shape and PG changes correlates with putative evolutionary events

To determine which genetic differences could be responsible for these changes, we screened for gene differences between genomes of species that diverged before and after node 1 and node 2 (excluding *N*. *wadsworthii* 9715). We used MycoHIT [16] as previously described. This software was designed to investigate the presence/absence of genes encoding orthologous proteins in conjunction with the phylogeny to finally detect horizontal gene transfers or deletion. To detect gene insertions, we used *N*. *meningitidis* proteins as the reference (database) that was compared by BLAST (TblastN) against all the other genomes. Similarly, for gene deletion, we used proteins from our complete genome of *N*. *elongata* as reference to search for orthologs in the other genomes.

For potential evolutionary events that could have helped the coccoïd transition and the first increase of GM5 at node 1, we were not able to find any horizontal gene transfers but we found a unique deletion of a gene annotated as *yacF*. This gene is absent in all bacteria that diverged after node 1 concomitantly with the appearance of the cell-shape change and it is present in all bacteria that diverged before node 1 except *N*. *wadsworthii* 9715 (Fig 1). Importantly, *yacF* (but not *mreBCD*, *pbpX*, *rodA* and *rodZ*) was also deleted from the coccus *N*. *wadsworthii* 9715 that could have undergone an independent cell-shape transformation.

Notably, as we detected a second increase of GM5 in the structure of the PG at node 2, we also screened for gene insertions and deletions that correlated with this event. We detected numerous genes deletions (7) and insertions (22) including several genes of unknown function (S2 Table). Hence some of them may be directly involved in the PG structural change observed. Interestingly, among all the events observed at this node, we detected the deletion of the elongation machinery (*mreBCD*, *pbpX*, *rodA*, *rodZ*) (Fig 1).

### *yacF* is almost exclusively present in bacilli

**Fig 3:**
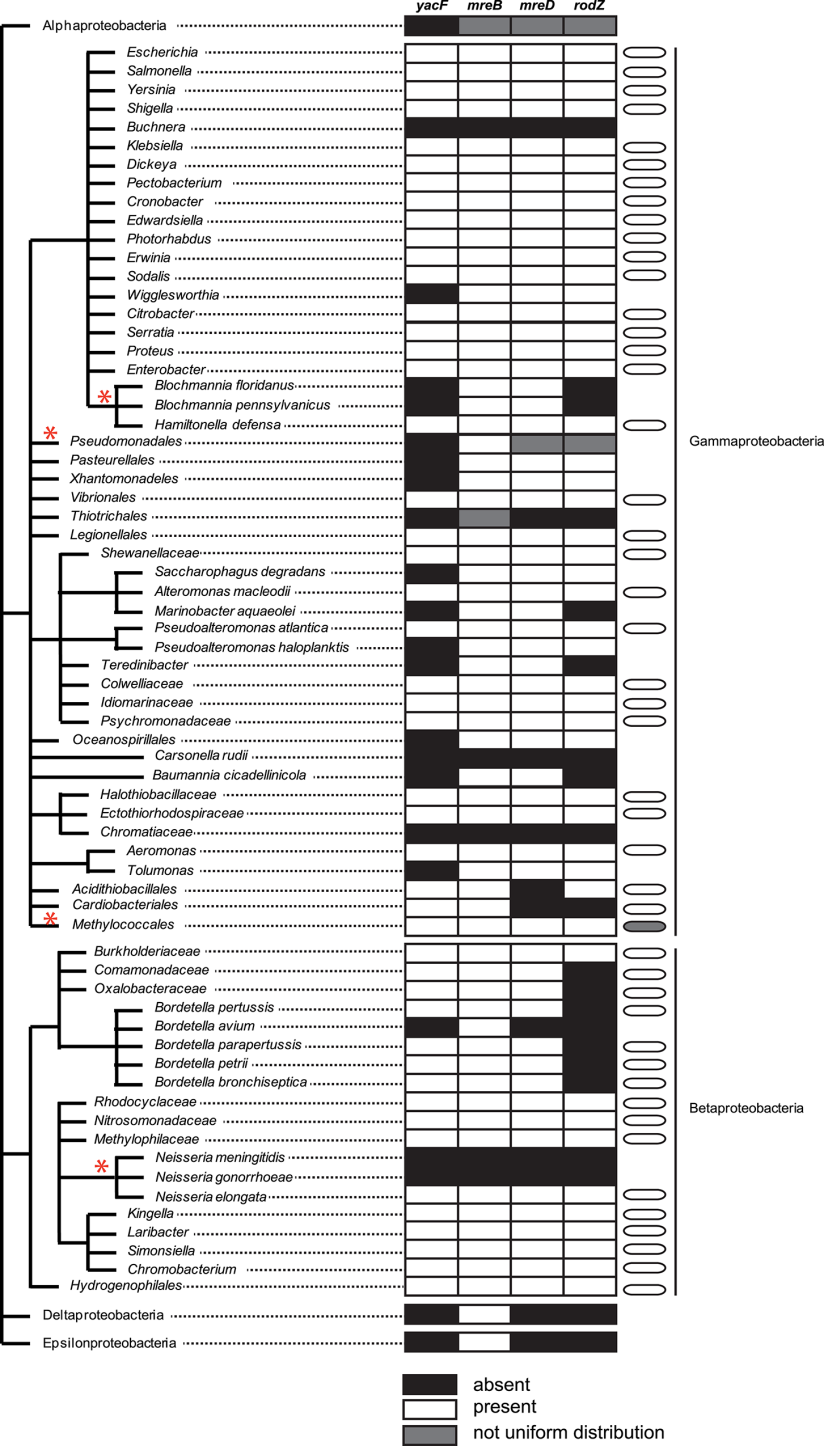
Distribution of *yacF* and other key components of the elongation machinery in proteobacteria. Representation of the proteobacteria taxonomy associated with a table presenting the information of the presence in all (white), absence in all (black). The presence/absence was assessed using the STRING database as previously described [45]. A grey box indicates that the distribution was not uniform (presence of outliers) in the specific lineage. Finally, the cell morphology is also presented only for *yacF*-positive lineages to emphasize that the majority of *yacF*-positive strains are bacillus. A grey cell-shape indicates the presence of outliers in the lineage (herein *Methylococcus capsulatus*). Red asterisks indicate situation where the distribution is described more in details in the text. (e.g. the case of *Pseudomonadeles* is described more in detail in the text and in the Fig 9).

We found only one evolutionary event that correlated with cell shape change at node 1, the deletion of *yacF*. Interestingly, this gene encodes a protein, YacF (or ZapD) that has been recently shown to be implicated in the *E*. *coli* cell cycle. It is localized at the midcell in a FtsZ-dependent manner [17]. In addition, *in vitro* data showed that the interaction of YacF with FtsZ promotes the bundling of FtsZ protofilaments [17]. Based on these results, it was suggested that YacF, *in vivo*, promotes FtsZ-ring assembly in *E*. *coli* [17]. Thus we hypothesized that the natural deletion of *yacF* in the ancestor of coccus *Neisseria* could have participated in the observed cell shape transition. Consequently, we investigated the correlation between the presence of *yacF* in bacterial genomes and the cell morphology (Fig 3). We observed that *yacF* is only present in β-proteobacteria or γ-proteobacteria and its presence is strongly correlated with bacilli cell shape. All the species harboring *yacF* are bacilli and inversely no coccoid bacteria harbor *yacF*. There is one exception to this rule in the *Methylococcales* order. This order has genera such as *Methylomonas* and *Methylomicrobium* that are bacilli and *Methylococcus*, which are cocci. The genome of the coccus *Methylococcus capsulatus* contains *yacF* but also all the elongation machinery genes (*mreBCD*, *pbpX*, *rodA*, *rodZ*). The cell cycle of methanotrophs is clearly less studied, and it remains to be determined how elongation and division is regulated in this bacteria. Finally, if *yacF* is almost exclusively present in bacilli, it is important to notice that some bacilli (such as *Xhantomonadeles* or *Pasteurellales*) do not harbor *yacF*. This is also the case for other accessory partners of the elongation machinery (such as MreD in *Acidithiobacillales* and RodZ in *Bordetella sp*.) (Fig 3). Overall, this distribution suggests an important role of YacF in bacilli

### Reconstruction of the ancestral *yacF* deletion in *Neisseriaceae* bacilli

**Fig 4:**
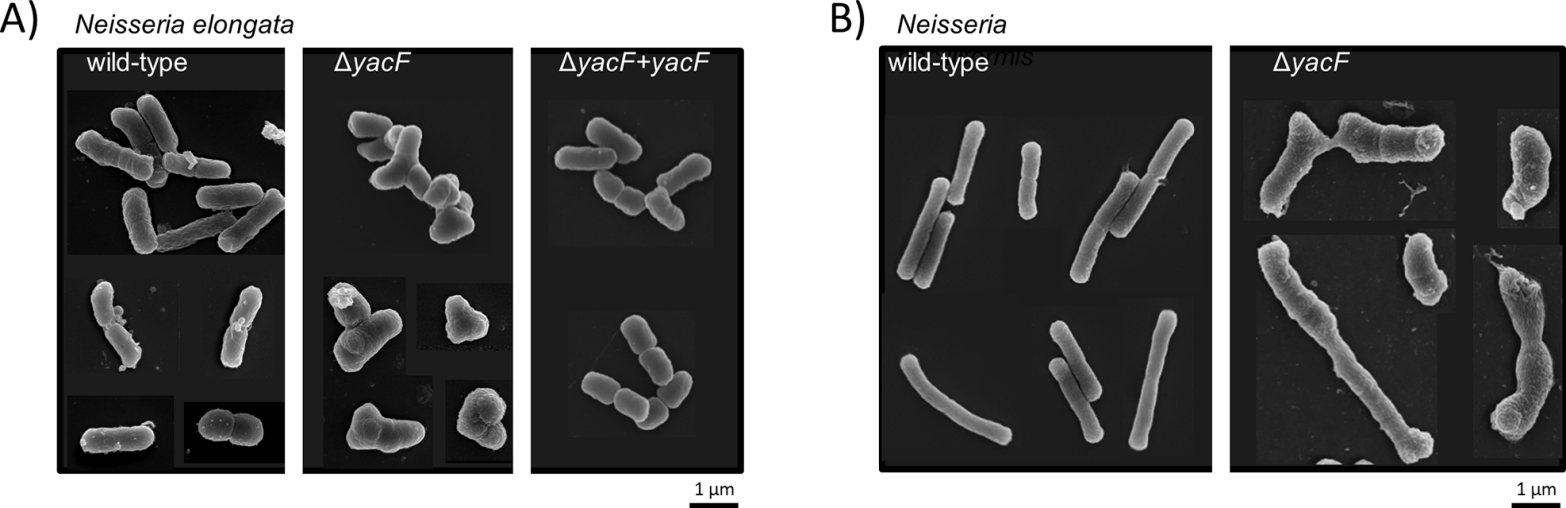
Effect of *yacF* deletion in *N*. *elongata* and *N*. *baciliformis*. Scanning electron microscopy images of A) *N*. *elongata* wild type, Δ*yacF* and ∆*yacF* complemented with *yacF* in a heterologous locus (Δ*yacF+yacF*) and B) *N*. *bacilliformis* wild type and Δ*yacF*.

As we noticed that *yacF* was present only in bacilli, but not in cocci, we hypothesized that its role may be restricted to bacteria with rod cell shape. To gain insight into its function, we deleted the gene in two elongated *Neisseria*: *N*. *elongata* (Fig 4A) and *N*. *bacilliformis* (Fig 4B). The ORF of *yacF* is potentially part of a conserved operon that includes *yacG*, a DNA gyrase inhibitor, *coaE*, a dephosphocoenzyme A kinase, and genes encoding proteins implicated in type IV pili production (*pilDFG*) (Fig 1). To exclude polar effects, due to *yacG* split-up from the rest of the operon, we developed an alternative genetic approach for *N*. *elongata*. We fused the start codon of *yacG* with the ATG of *yacF*. This permitted to produce a non-interrupted operon that resulted in the control of *yacG* by the putative native promoter of the operon. In this condition, a similar expression of *yacG* was measured using RT-PCR for the *N*. *elongata* wild type and the Δ*yacF* strains (S1 Fig) therefore, excluding a potential role of YacG in phenotypes observed. When *yacF* was deleted, we noticed strong morphological defects for all cells observed in both species (*N*. *elongata* and *N*. *bacilliformis*) (Fig 4) with a multitude of morphological aberration from multipolar cells, to abnormally elongated cells. These defects were restored by complementation with *yacF* in *N*. *elongata* (Fig 4A). This important morphological defect was accompanied by a moderate reduction of the growth rate (S2 Fig).

**Fig 5:**
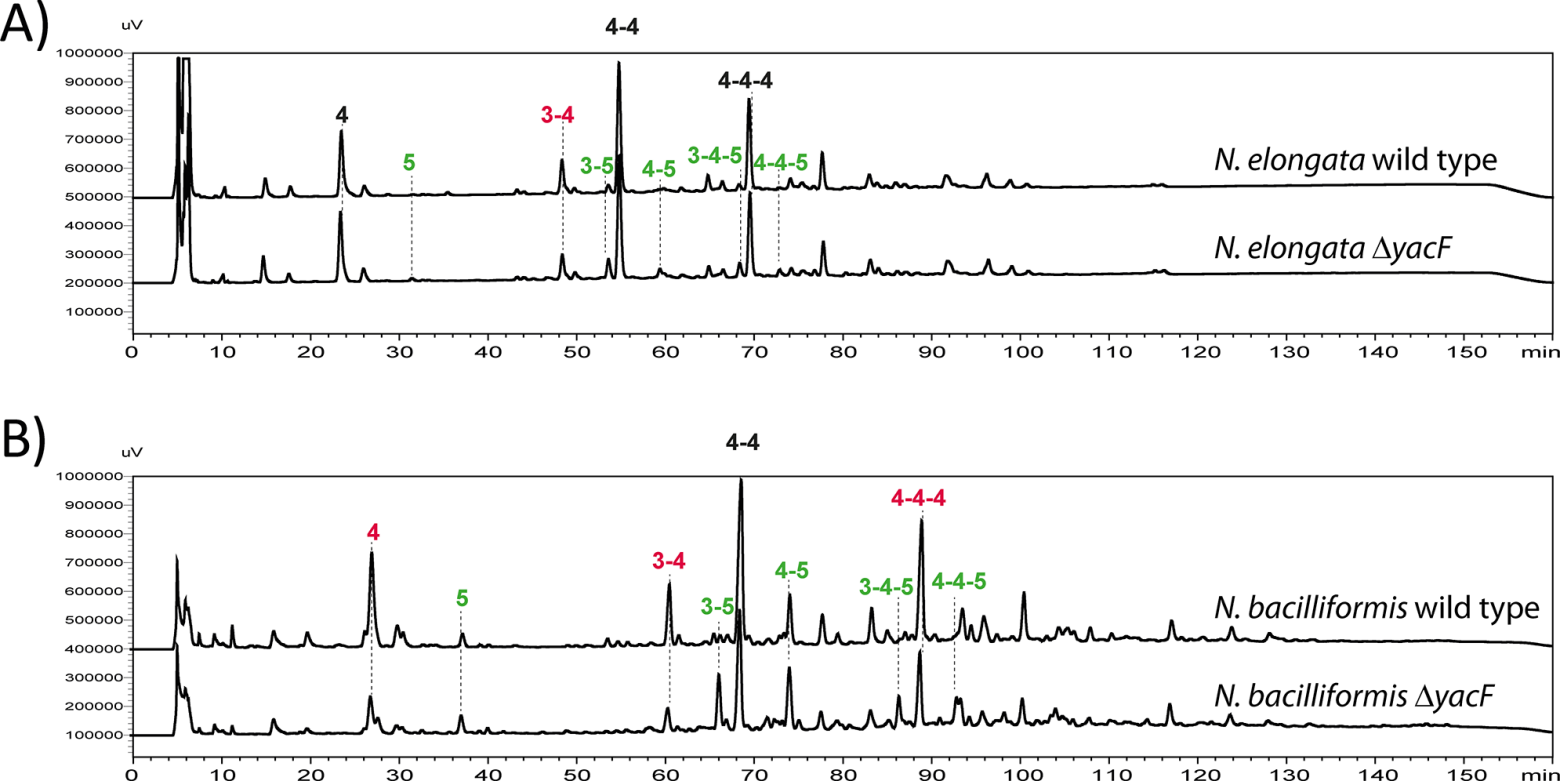
Increased proportion of pentapeptide muropetides in absence of YacF. Reverse-phase HPLC analyses of muropeptides after mutanolysin digestion of purified insoluble PG of: A) *N*. *elongata* wild type and Δ*yacF* and B) of *N*. *bacilliformis* wild type and Δ*yacF*. The numbers indicate the different muropeptides; for example, 3 stand for GM3. The peak labeled 4–4 corresponds to a dimer of GM4 crosslinked by the two stem peptides. The colors indicate a, more than two fold, increase (green) or decrease (red) of the indicated muropeptide in the Δ*yacF* PG. This is representative chromatogram of experiments done at least three times. The raw quantification can be found in S3 Fig for *N*. *elongata* and the corresponding mutants.

To verify if this aberrant morphology was linked to cell wall synthesis, we measured the muropeptides content of the PG extracted from these mutants using reverse-phase HPLC (Fig 5). The *yacF* mutant in both *N*. *elongata* and *N*. *bacilliformis* showed an increase of muropeptides composed of pentapetides (GM5) and a decreased of tetra-peptides (GM4) compared to wild-type bacteria (Fig 5) as observed during natural deletion of *yacF* at node 1 (Fig 1). The quantification of these differences in *N*. *elongata* is also presented in S3 Fig.

### Morphological aberration due to the *yacF* deletion can be compensated by a lack of cellular elongation or division

In *E*. *coli* YacF has a FtsZ-dependent mid-cell localization, that takes place before the septum formation [17]. As we observed abnormal elongation and abnormal division in *N*. *elongata* and *N*. *bacilliformis* lacking *yacF*, we hypothesized that this protein may play a role in regulating the transition between elongation and division at the mid-cell of elongated *Neisseria*. If this is true, in the absence of one of these events the role of YacF may be minimal.

**Fig 6:**
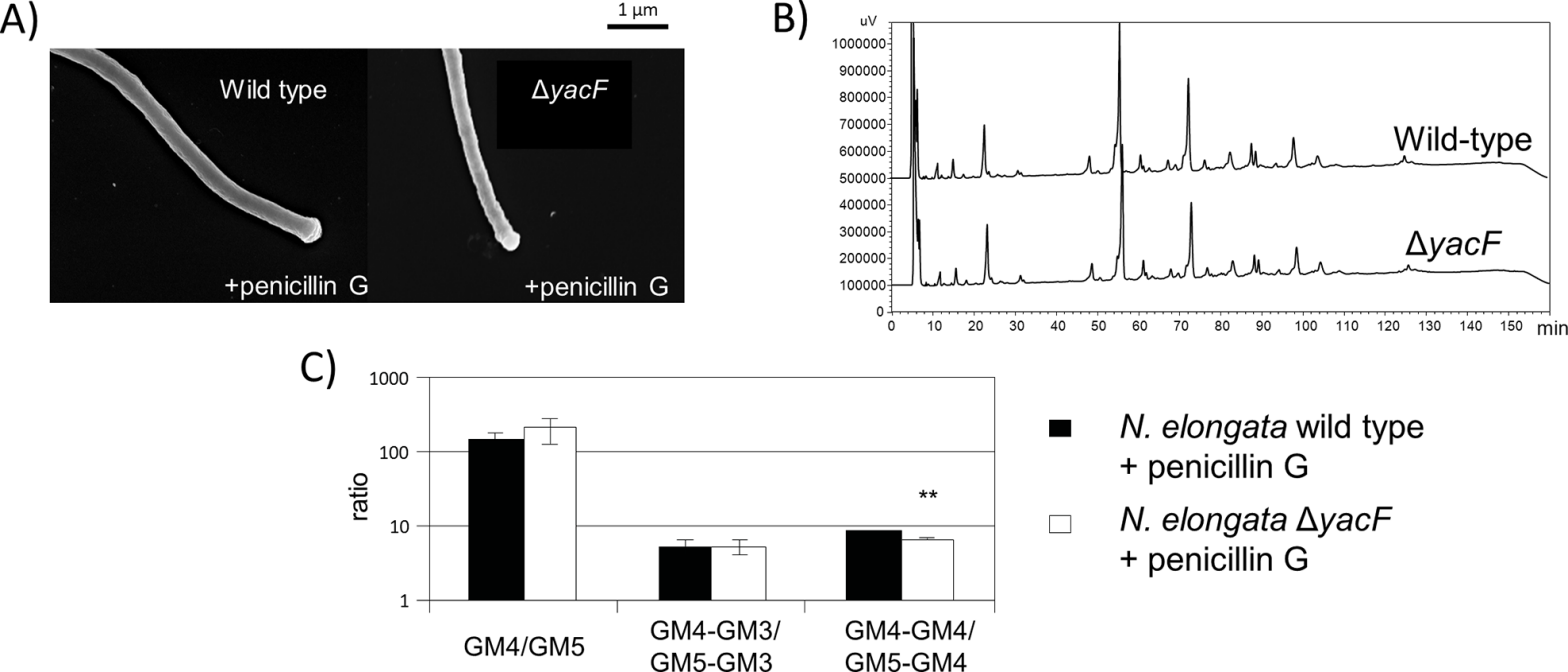
Deleterious effects of *yacF* deletion can be fixed by inhibiting cell division. A) Scanning electron microscopy images showing similar morphology (filaments) of *N*. *elongata* wild type and Δ*yacF* grown in presence of sub-inhibitory concentrations of penicillin G. B) Similar reverse-phase HPLC profile of muropeptides after mutanolysin digestion of purified insoluble PG of *N*. *elongata* wild type and Δ*yacF* grown in presence of penicillin G. C) Similar ratio of GM4 over GM5, GM4-GM3 over GM5-GM3 and finally GM4-GM4 over GM5-GM4. (*** p≤0.001; ** p≤0.01; * p≤0.05 compare to wild-type).

To test for the role of YacF in absence of division, we used penicillin G. This antibiotic targeting PBPs, used at an optimal concentration close to the inhibitory dose, blocks cell division but not elongation [18]. This consequently leads to the filamentation of *N*. *elongata* cells [18] by inhibiting specifically PBP2 but not PBPX. We used this property and assessed for potential differences in morphology (Fig 6A) or PG structure (Fig 6B) as the ratio between GM4/GM5 (Fig 6C). We observed no differences between the wild type strain and its *yacF* mutant when the division was inhibited.

**Fig 7:**
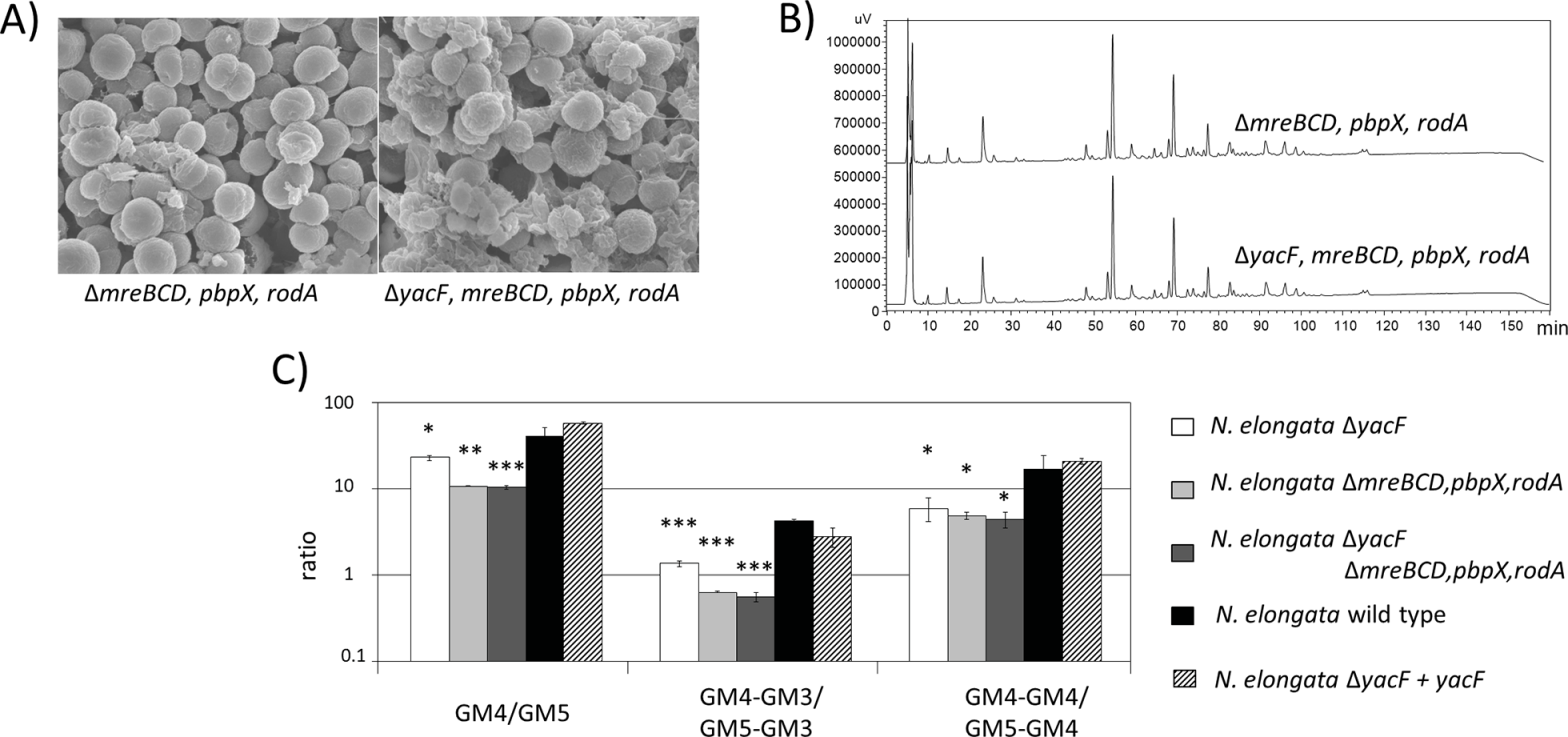
Deleterious effects of *yacF* deletion can be fixed by inhibiting cell elongation. A) Scanning electron microscopy images showing similar morphology (coccus) of *N*. *elongata* Δ*mreBCD*,*pbpX*,*rodA* and the double mutant Δ*yacF* Δ*mreBCD*,*pbpX*,*rodA*. B) Same reverse-phase HPLC profile of muropeptides after mutanolysin digestion of purified insoluble PG of *N*. *elongata* Δ*mreBCD*,*pbpX*,*rodA* and the double mutant Δ*yacF* Δ*mreBCD*,*pbpX*,*rodA*. C) Similar ratio of GM4 quantity over GM5, GM4-GM3 over GM5-GM3 and finally GM4-GM4 over GM5-GM4 in the PG of Δ*mreBCD*,*pbpX*,*rodA* and the double mutant Δ*yacF* Δ*mreBCD*,*pbpX*,*rodA*. The raw quantification can be found in S2 Fig for *N*. *elongata* and the corresponding mutants. The ratio of wild type bacteria, the single mutant Δ*yacF* and the complemented strain Δ*yacF+yacF* are also presented. The increased proportion of GM5 in the PG of Δ*yacF* (as shown in Fig 3) can be again observed but this increase is less important than when the *mreBCD*,*pbpX*,*rodA* locus, encoding for the elongation machinery, is deleted. (*** p≤0.001; ** p≤0.01; * p≤0.05 compare to wild-type).

To test the role of YacF in absence of elongation, we constructed a strain deleted from the entire *mreBCD*,*pbpX*,*rodA* locus and assessed for potential differences in morphology (Fig 7A) or PG structure (Figs 7B and S2) as the ratio between GM4/GM5 (Fig 7C) correlated with the absence of *yacF*. We observed neither aberrant morphological problems nor PG composition differences between the mutant Δ*mreBCD*,*pbpX*,*rodA* and the double mutant Δ*yacF*; Δ*mreBCD*,*pbpX*,*rodA* (Fig 7A, 7B and 7C). All together our data shown that YacF is not required in absence of the elongation or the division for normal morphology or PG structure suggesting that YacF is implicated in the coordination/transition between these two events.

### Reconciling *in vitro* deletion with natural *Neisseriaceae* evolution

As seen in Fig 7C, wild type *N*. *elongata* (a species that diverged before node 1) has a ratio GM4/GM5 (or GM4_GM4/GM4_GM5) superior to Δ*yacF* (representation of bacteria that diverged after node 1 and before node 2) that has itself a ratio superior to the double mutant Δ*yacF*; Δ*mreBCD*,*pbpX*,*rodA* mutant (representation of bacteria that diverged after node 2) (Fig 7C). Thus, by reconstructing two different genetic events associated with node 1 and 2, we were able to recapitulate the step-wise change in the PG structure observed in *Neisseriaceae*.

Based on the suggested role of YacF, in regulating the transition between elongation and division and the well-characterized role of MreBCD, PBPX, RodA, RodZ in elongation, it was surprising to notice that during evolution *yacF* deletion occurred prior to that of the elongation machinery. To better understand this evolutionary time-lapse, we compared the natural evolution to the alternative expected order of events, i.e. prior deletion of the elongation machinery. Thus, we compared the growth fitness of the wild type *N*. *elongata*, the simple mutant Δ*yacF* or Δ*mreBCD*,*pbpX*,*rodA* and the double mutant Δ*yacF*; Δ*mreBCD*,*pbpX*,*rodA*. The Δ*mreBCD*,*pbpX*,*rodA* showed a strong growth defect that can be partially complemented by *yacF* deletion (S2 Fig). This suggests that deletion of *yacF* was prior to the elongation machinery to reduce the fitness burden of changing cell shape.

### Adaptation in response to cell-shape changes

**Fig 8:**
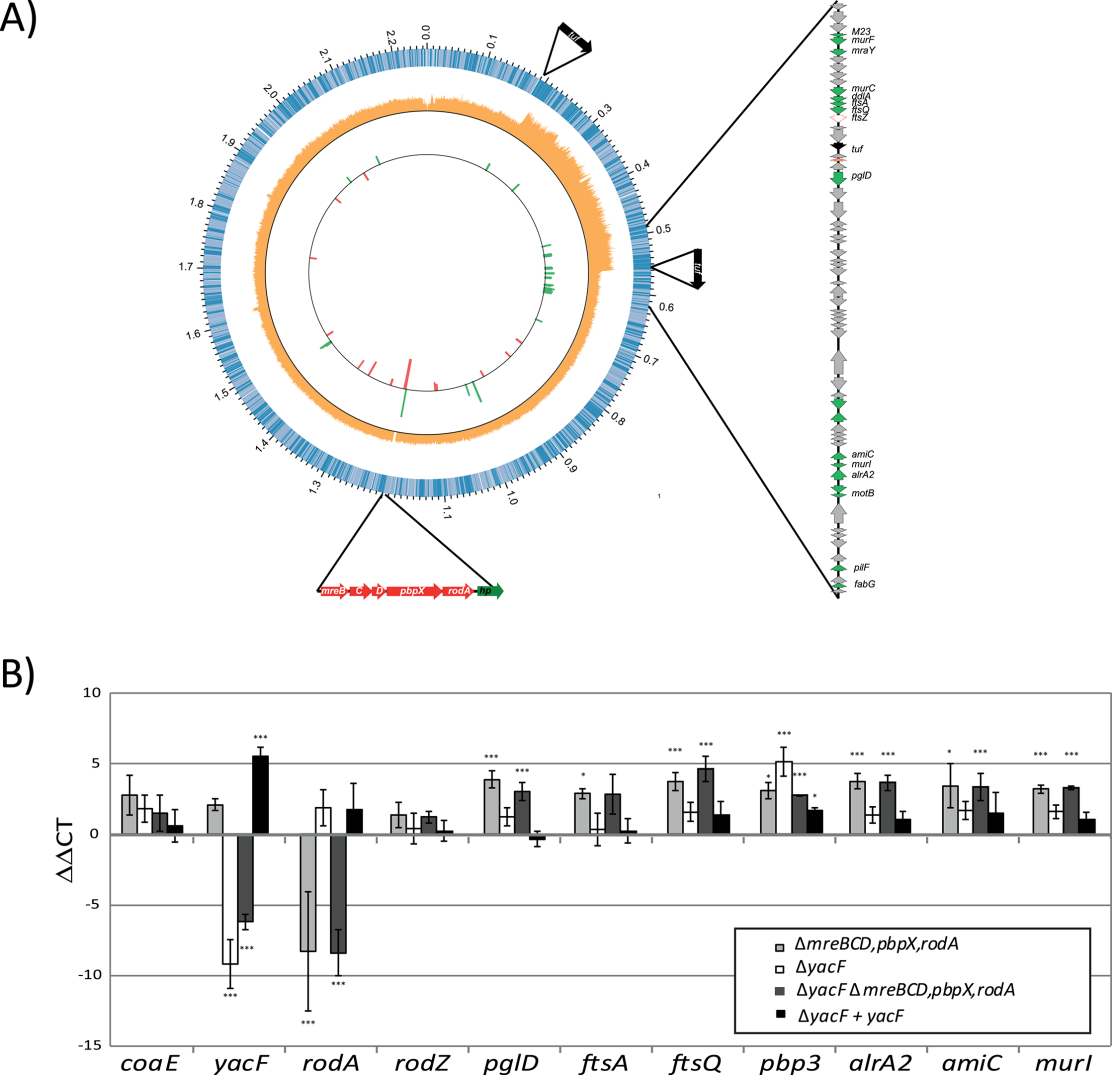
Cellular adaptation to artificial coccoïd transition. A) A graphical representation of the *N*. *elongata* genome sequenced by PacBio is illustrated here using CIRCOS software. The first circle represents the different *orf* and their orientation. The second circle represents the coverage values (orange) of the Illumina reads obtain by sequencing *N*. *elongata* Δ*mreBCD*,*pbpX*,*rodA* and mapped along the genome of *N*. *elongata* wild-type. One can notice the increased coverage by two fold of the DNA region from 0.202 to 0.558 Mbp. Finally, the third internal circle represent the fold change expression, in function of the position, of genes up-regulated (green) and down-regulated (red) of Δ*mreBCD*,*pbpX*,*rodA* compare to wild type *N*. *elongata*. Only genes with a p-value below 0.01 and fold change over 3 are represented. All the fold change values and statistics are presented in S3 Table. B) Verification of transcriptional changes of selected genes, in the different mutants, using rt-PCR and calculated using ΔΔCT (*** p≤0.001; ** p≤0.01; * p≤0.05).

The deletion of the elongation machinery was difficult to achieve in *Neisseriaceae*. We were unsuccessful in deleting the locus in *N*. *bacilliformis* and we obtained only one clone after several assays in *N*. *elongata*. This clearly suggested a need for bacterial adjustment to lose the elongation machinery. We reasoned that this *in vitro* evolution could reveal some additional genetic changes (for example, suppressor mutations) that might have also occurred naturally during the evolution of the *Neisseriaceae* familly. Therefore, we Illumina-sequenced the genome of the *N*. *elongata* Δ*mreBCD*,*pbpX*,*rodA* and compared it to our complete wild-type *N*. *elongata* PacBio-sequenced genome. We identified only two SNPs: 1) position 2052208 in *ccoN*. It is a frameshift mutation that restores *ccoN*. As *ccoN* is not a pseudogene in the other *N*. *elongata* sequence, we concluded to a sequencing error in our wild-type bacteria genome. 2) position 3614 in NELON_00025; a pseudogene encoding an insertase. These two SNPs are unlikely to be linked to a physiological adaptation to the cell shape change. Besides these two SNPs, we also observed a duplication of a 350kb region that is surrounded by two repeated *tuf* genes (Fig 8A). Interestingly, the region encompasses the *dcw* (division cell-wall) cluster and ends with *ftsZ* the major actor of cell division. We hypothesized that this duplication, although without SNPs, could affect gene expression by changing the chromosome structure. We performed RNA sequencing analyses (Fig 8A) and qRT-PCR validation (Fig 8B) on RNA from *N*. *elongata* strains harboring deletions of *mreBCD*,*pbpX*,*rodA* compared to the wild-type strain. The effect of the *mreBCD*,*pbpX*,*rodA* deletion on gene expression was reproducible between the single and double mutants and mainly restricted to genes in the region surrounding the second *tuf* gene around 0.5 Mb (Fig 8A). The duplication of this 350 kb region lead to the specific up-regulation of genes linked with cell wall remodeling such as *murF*, *murC*, *murI*, *ftsA*, *ftsQ*. This strongly suggests that during *in vitro* evolution, the division machinery needed to be over-expressed in order to compensate for the loss of the elongation.

### Similar evolution observed for another nasopharyngeal pathogen

**Fig 9:**
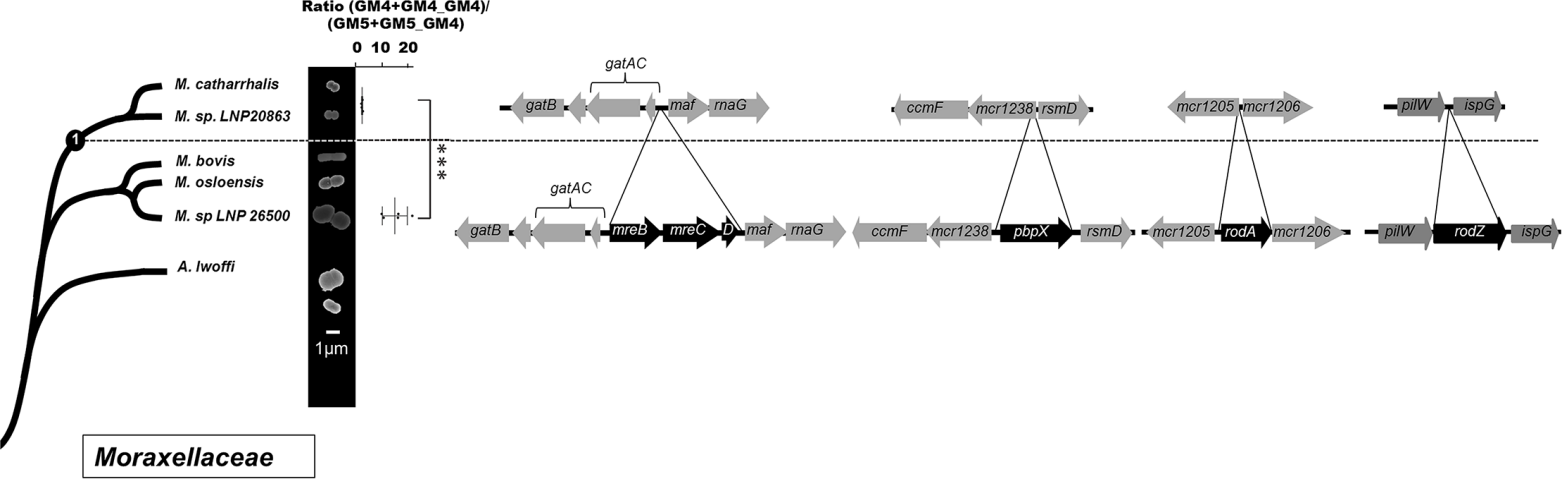
Cell shape and PG structure evolution among the *Moraxellaceae* family. Schematic phylogeny of the *Moraxellaceae* family, based on 16s analysis, along with scanning electronic microscopy images of representative species. The mean ratio (GM4+GM4_GM4)/(GM5+GM5_GM4) is presented (with standard deviation) for the different lineages (*** p≤0.001; ** p≤0.01; * p≤0.05). Each dote, represents an independent isolate (tested for *pbpX* presence) from the CNRM collection that have been classified as *M*. *catharrhalis* (3), *M*. *sp LNP20863* (1), *M*. *bovis (gift from Dr*. *S*. *Higlander—1) M*. *osloensis* (1), *M*. *sp*. LNP 26500 (1), *A*. *lwoffi* (2). Finally, the right part displays the deletion detected at the node of evolution by presenting the genomic organization of species that diverged before node 1 (assessed from the genome sequence of *M*. *bovis* and *A*. *lwoffi*) and after node 1 (from the *M*. *catharrhalis* genome).

We showed that the deletion of *yacF* that encodes a putative coordinator of the elongation and division, was one of the starting points of the coccoïd transition in *Neisseriaceae*. This is also true for other bacteria, such as the coccoïd endosymbiote *Blochmania sp*. that lost *yacF* but preserved other components of the elongation machinery (as *mreBCD* see Fig 3). Additionally in the pseudomonadales order composed of the well-known *Acinetobacter* and *Moraxella* genera, all sequenced bacteria lack *yacF* suggesting a common ancestral deletion (Fig 3). Similar to the *Neisseriaceae* family, *Moraxellaceae* also comprises species with different cell shape varying from rod to coccobacilli to cocci (*M*. *catarrhalis* species). By using the same approach described above, we observed that this cell shape change was also correlated with the family phylogeny (Fig 9). In addition, as observed for *Neisseriaceae*, the coccobacillus-to-coccus transition was concomitant with a decrease in the GM4 proportion over the GM5 in the PG composition measured by (GM4+GM4_GM4)/(GM5+GM5_GM4) ratio. This coccus transition was concomitant with the deletion of *mreBCD*, *pbpX*, *rodA*, *rodZ*, (Fig 9).

### First clues about the advantage of the rod-to-cocci evolution

**Fig 10:**
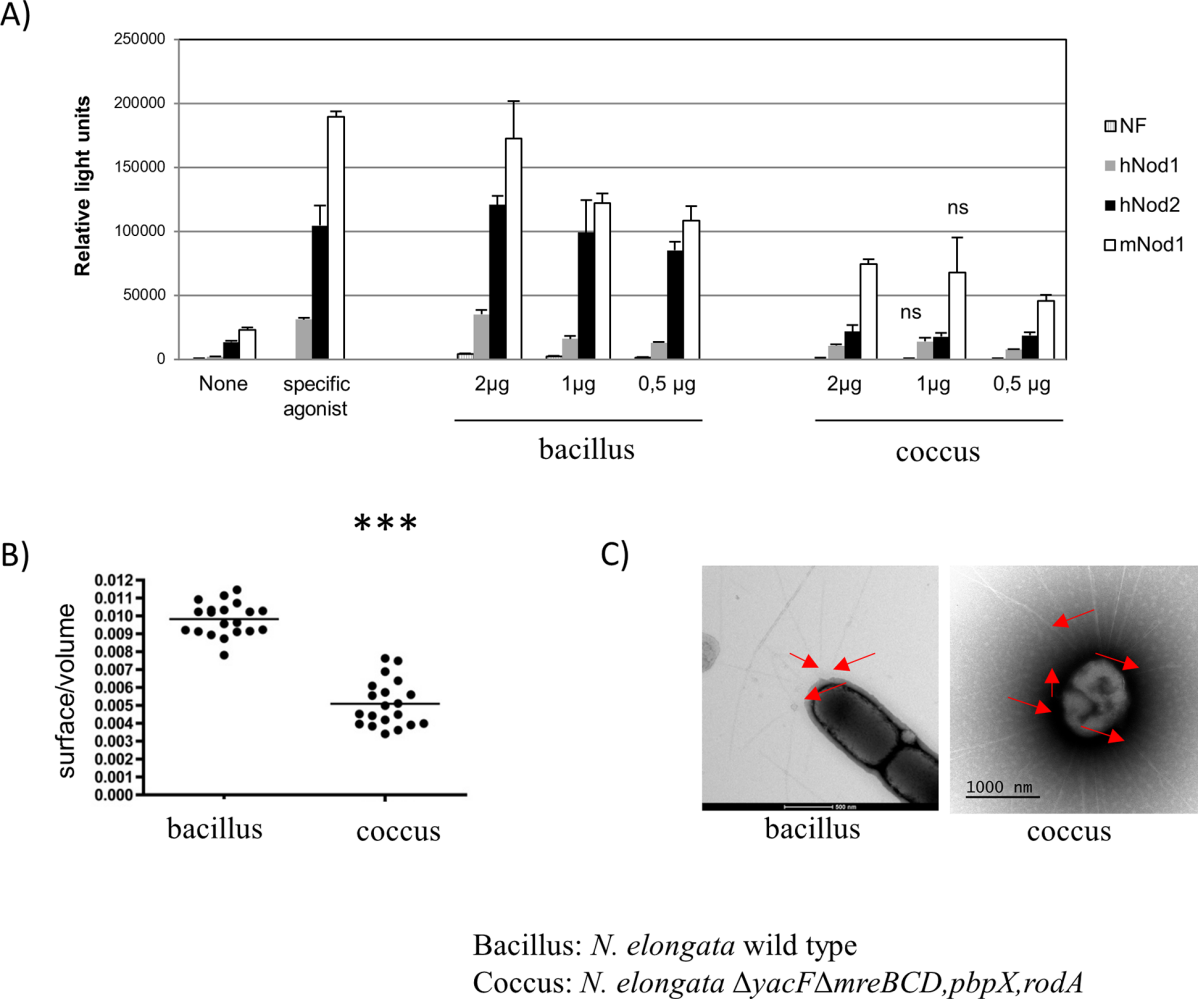
Different properties of coccus cells. A) NF-κB luciferase expression in response to PG from *N*. *elongata* rod (wild-type) or cocci (Δ*mreBCD*,*pbpX*,*rodA* Δ*yacF*) of HEK-293 cells transfected with human Nod1 (grey), human Nod2 (black) and murin Nod1 (white). The two controls measured the luciferase in absence of stimulation or in presence of purified specific agonist (MurTriDap for hNod1, MDP for hNod2, and MurTetraDap for mNod1). This is representative result of two independent experiments. All the results are statistically significant (p<0.05 rod vs cocci) except those noted ns (for non statistically significant). B) Estimated ratio surface/volume extracted from SEM images of around 20 cells (*** p≤0.001) and C) TEM image showing pili (red arrow) from *N*. *elongata* wild type (bacillus) and *N*. *elongata* Δ*yacF* Δ*mreBCD*,*pbpX*,*rodA* (coccus).

Twice during the evolution of nasopharyngeal symbiote, we observed an evolution that ends with a cell shape change and a modification of the structure of the PG. It was recently shown that changes in the *N*. *meningitidis* PG composition affects the bacterial detection by dedicated host receptors [19]. It is therefore possible that the changes observed during the evolution toward *N*. *meningitidis* will also affect the host-pathogen relationship by affecting the Nod1 and Nod2 recognition. To test this, we measured NF-κB luciferase expression in response to PG recognition by Nod proteins using previously described assay [20, 21]. The Nod1 and Nod2 responses to *N*. *elongata* (bacillus) purified sacculi were superior to the response of the double mutant sacculi (coccus) (Fig 10A) suggesting that the coccal PG, exclusively composed of polar/septal PG, is a less potent Nod activator than the bacilli *in vitro*.

Except from the host-pathogen interaction, we also hypothesized that these changes may have other unsuspected impacts important for colonization of the NP. First of all, using SEM images, we estimated the volume and the surface of bacteria cells of wild-type *N*. *elongata* (bacillus) and the double mutant (coccus) and calculated the ratio surface/volume (Fig 10B). Our results showed that the coccoïd cell shape has the property to present a reduced cell surface over the intracellular volume.

Finally, the absence of elongation led to cells composed of PG produced by PBP2 (septal/polar PG, enriched in GM5) and a complete absence of lateral PG. We hypothesized that the presence of GM5 in the septal/polar PG may be a marker of localization of some cell apparatus. As such, a structure that localized preferentially to the poles in diplo-bacilli (wild type *N*. *elongata*) would be localized all over the cell in the diplo-cocci (*N*. *elongata* Δ*mreBCD*,*pbpX*,*rodA;* Δ*yacF*). Using negative contrast TEM, we studied pili localization in the two types of isogenic bacteria. We observed a clear preference for polar localization of this apparatus in *N*. *elongata* (Fig 10C) while it was evenly distributed all over the cell in the double mutant *∆yacF;∆mreBCD*,*pbpX*,*rodA*. This result indicates that the observed differential localization of pili between *N*. *elongata* (polar/septal) and coccoïd *Neisseria* as *N*. *meningitidis* or *N*. *gonorrhoeae* (all-over the cell) is linked to the coccoïd transition that took place during the emergence of these species.

## Discussion

*N*. *meningitidis* and *M*. *catarrhalis* are two bacteria that share the same ecosystem as proved by recent horizontal genes transfer between these species [22]. The present work establishes that the ancestor of those bacteria has undergone the same cell shape transformation from a rod to a coccus accompanied by a PG enrichment in GM5. Our evolutionary approach highlighted the central role of *yacF* as a starting point of the coccus transition for several bacilli. Importantly, this approach allows description of the role of YacF (ZapD), in the coordination of the elongation and division. This work is reminiscent of the recent description of YacF in *E*. *coli* [17]. Durand-Heredia and colleagues described a link between YacF and FtsZ. However, they were not able to establish a role for YacF as *yacF* deletion has no effect on cell morphology in *E*. *coli*, suggesting a redundancy with unknown YacF functional homologues. This is also strengthened by the fact that some bacilli lack homologues of the *yacF* gene.

When the deletion was reconstructed in *N*. *elongata*, we were not able to completely mimic the evolution suggesting that some additional events and compensatory mutation are also necessary. We tried to reverse the evolution in *N*. *meningitidis* by re-introducing *yacF*, *rodZ*, *mreBCD*, *pbpX*, *rodA* but this had no effect on cell morphology or PG composition. This supports the notion that additional changes, independent of those genes, are necessary. It seems unlikely to involve defects of the *dcw* cluster (the cluster of division and cell wall synthesis) of *N*. *meningitidis* as it is similar to bacilli *dcw* cluster and not to cocci cluster [6, 23]. In contrast, it could involve an increased expression/activity of the divisome as suggested by the *in vitro* coccus evolution. Therefore, further studies will be necessary to completely understand the coccoïd transition by, for example, applying several rounds of *in vitro* passage of *N*. *elongata* Δ*yacF* to select for cell shape stable variants.

It was surprising to us not to observe the deletion of the elongation machinery associated with the cell shape transition in *Neisseriaceae*. Indeed several *Neisseria* cocci retain the machinery (Fig 1). This suggests a role of this machinery in cocci bacteria that may be independent of elongation. Indeed our PG structural data showed an intermediate GM4 over GM5 ratio compared to cocci that deleted the machinery. These results suggest that some components on the machinery are still involved in PG synthesis. It may be possible that their presence is still required to localize properly or organized temporally the divisome in bacteria that recently underwent cell shape transition. It has been shown in *E*. *coli* that some components of the elongation machinery interact with components of the divisome [24]. This hypothesis could explain the strong growth defect of the *mreBCD*,*pbpX*,*rodA* mutant in *N*. *elongata*, which correlated with an increased expression of some proteins of the divisome (AmiC, MurI, FtsA, FtsQ) and the failure to delete the same locus in *N*. *bacilliformis*. Evolution may have to first select events that will phenocopy the deletion of the elongation machinery (Δ*yacF*) to stepwise adapt the divisome (or other physiological processes) that still required the presence of elongasome proteins.

Two phylogenetically distinct bacteria specialized in the colonization of the NP underwent convergent evolution. Several hypothesis have been emitted to explain different cell shapes as these would be beneficial for motility, to compete for nutrient or to resist to predation and physical constraint (turgor pressure) among other [25–27]. *Kingella* sp. *Simonsiella* sp. *Eikinella* sp. and some *Neisseria* are often found in the oral cavity in the saliva, an aqueous environment, whereas *N*. *meningitidis* is attached to the NP mucosa a drier environment. One hypothesis may be that bacilli, with polar pili, are more adapted to move (twitching motility) in the saliva in contact with the buccal mucosa with directional movement [28] whereas cocci with pili all over the cell may be more adapted to attach the dryer nasopharyngeal mucosa. An alternative hypothesis is that the coccus is less susceptible to attacks from the immune system or bacterial killing systems (as Contact-Dependent Inhibition CDI system) as the ratio surface/volume is smaller than that of bacilli. In support for this hypothesis several studies has shown that cell surface size is a key factor when facing immune attacks [29, 30]. We therefore think this cell shape change may represent an advantage in the case of *N*. *meningitidis* in the NP to increase resistance to aggressions.

The increased proportion of GM5 in the PG of NP pathogens may also represent some advantages. Our group recently showed that, when *N*. *meningitidis* experiences changes in the PG composition, including a decreased ratio GM4/GM5, the recognition by the innate immune system is altered with less induction of the immune response [19]. In this work, we observe similar results using PG from engineered *N*. *elongata* coccus strain that harbors a decreased ratio GM4/GM5. It is therefore possible that the PG with increased proportion of GM5 is processed or recognized differently. In support of this, a study reported that ligand-induced structural rearrangements occurred in the PG-binding site of the human PGRP (PG recognition protein) co-crystalized with pentapeptide muropeptides but not with tripeptide muropeptides [31]. It can also be possible that an optimal ratio of GM5 over the GM4 is required for permissive interaction. Apart from a role in host innate immune system interaction, the presence of GM5 in the PG may also be a way to avoid PG bacteriolytic effectors, injected in the periplasm by neighboring bacteria, by altering their natural substrate [32].

In this work, we characterized the process of cell shape transition during bacterial evolution within a defined ecological niche by highlighting some of the genetic events that have been selected as well as the factors and advantages that may have driven this selection. Using an evolutionary approach, we added a new layer of understanding of the function of a protein involved in coordinating cell elongation and division. Understanding the fitness of these pathogens to the NP and their survival and their invasive capacity may help tailoring intervention strategies to favor asymptomatic carriage and to prevent invasive life-threatening infections

## Materials and Methods

### Bacterial strains and culture conditions

All *Neisseria* were grown in GCB agar medium with Kellogg supplements. For cloning experiments, *E*. *coli* DH5α was grown at 37°C in Luria-Bertani Media (Difco). When required, antibiotics were added as follows: chloramphenicol (30 μg/ml for *E*. *coli*; 5 μg/ml for *Neisseria* sp.), kanamycin (50 μg/ml for *E*. *coli*; 100 μg/ml for *Neisseria* sp.) and erythromycin (300 μg/ml for *E*. *coli*; 3 μg/ml for *Neisseria* sp.) and sub-inhibiting concentration of penicillin G (0.12 μg/ml). *N*. *elongata* subsp. *glycolytica* (29315), *N*. *lactamica* (23970), *N*. *bacilliformis* (BAA-1220), *N*. *sicca* (29256), *K*. *oralis* (51147) were purchased at the American Type Culture Collection (ATCC). *E*. *corrodens* and *M*. *bovis* were obtained from Dr. Gaillot and Dr. S. Highlander, respectively. Other strains were from the collection of Centre National de Reference des Meningocoques (CNRM, Institut Pasteur, Paris).

### Mutants construction

To generate the deletion of *mreBCD*, *pbpX*, *rodA* in *N*. *elongata*, we first amplified the surrounding region in 5’ or 3’ with the respective couple of primers 5’RD1NeF-5’RD1NeR and 3’RD1NeF-3’RD1NeR. We ligated the 5’ fragment digested with XmaI and XbaI into the plasmid pCom-Pind [33]. The resulting plasmid was digested with BamHI and SpeI and ligated with the 3’ fragment digested with the same enzymes to generate plasmid p3’KORD1Ne3’::Cm.

To generate the *yacF* deletion in *N*. *elongata*, we first amplified the upstream 5’ region of *yacF* with the primer set 5’RD2NeF-5’RD2NeR. This PCR fragment was digested with NsiI and PstI and ligated into the plasmid pGEM::Km [34] digested with NsiI to generate p5’RD2Ne::Km. *yacG* was amplified using primer set YacGF-YacGR and fused to the rest of the operon by inserting the PCR fragment digested with MluI and SpeI to p5’RD2Ne::Km digested with the same enzymes. The resulting plasmid was called p5’RD2GNe::Km. The construct along with the kanamycin resistance cassette was amplified using 5’RD2NeF-KmXbaIR primer set and fused to the downstream 3’ region of *yacG* amplified with 3’RD2NeMoinsF-3’RD2NeR. To achieve this fusion, we used a multi-step PCR reaction with the two PCR product as template and 5’RD2NeF-3’RD2NeR as primers. The resulting 3kb PCR product was named 5’RD2GNe3’::Km.

To complement the deletion of *yacF* in *N*. *elongata in trans*, we constructed a plasmid that allows insertion in an intergenic region at the end of *nrqF*. We amplified 5’ and 3’ regions surrounding the target locus of insertion with 5nrqF-5nrqR and 3nrqF-3nrqR primer set, respectively. We ligated the 5’ fragment digested with SpeI and BamHI with the plasmid pCom-Pind [33]. The resulting plasmid was digested with XbaI and NcoI and ligated with the 3’ PCR fragment digested with the same enzymes to generate p5nrq3::Cm. In parallel, the firefly luciferase orf was inserted into pBAD28 (Life Technologies) using NcoI and PstI to generate pBAD::*luc*. A *Neisseria pilE*p promoter was amplified from *N*. *meningitidis* MC58 DNA using pilEpF-pilEpR primers and cloned in front of the luciferase genes using NcoI and NheI in pBAD::*luc* to generate p*pilE*pLuc. In this plasmid, luciferase was subsequently replaced by *yacF* that has been amplified using yacFNcoIF-YacFPstIR primer set and digested with NcoI-PstI to generate p*pilE*p*yacF*. Finally the cassette containing *pilE*p and *yacF* was excised using NheI and PstI, treated with T4-polymerase (Fermentas) and inserted into p5nrq3::Cm that was digested with BamHI and blunt-ended with T4-polymerase to generate the final plasmid pcomp*YacF*::Cm.

To generate the *yacF* deletion in *N*. *bacilliformis*, we first amplified the 5’ upstream or 3’ downstream region with the respective couple of primers 5’KORD2NbF-5’KORD2NbR and 3’KORD2NbF-3’KORD2NbR, respectively. We first ligated the 3’ fragment digested with NcoI and SphI into the plasmid pGEM::Km [34]. The resulting plasmid was digested with NsiI and ligated with the 5’ digested with NsiI and PstI to generate the plasmid p5’KORD2Nb3’::Km.

Bacteria were transformed with linearized plasmids or PCR products for 5’RD2GNe3’::Km as followed: bacteria were inoculating on a GCB agar plate and 10 μl of around 500 ng of DNA was deposed on top of the culture. After an overnight incubation, bacteria were collected and inoculated in selective GCB agar plates containing the corresponding antibiotics.

### PG preparation

Bacteria were inoculated on GCB agar plates without antibiotics (except in the case of the penicillin G treatment experiments) and incubated at 37°C in a 5% CO2 atmosphere during 16h. PG was isolated by an adapted version of the method developed for *E*. *coli* [35] with all steps carried out at a pH below 7.0. Bacteria were collected and dispersed in 10 ml of cold distilled water (pH 6.0). The cells were added drop-wise to 10 ml of boiling 8% SDS buffered and boiled for a further 30 min. After cooling to room temperature overnight, the SDS-insoluble material was collected by centrifugation at 39000 × g for 30 min. The pellet was washed seven times with warm distilled water (pH 6.0), lyophilized, resuspended to a final concentration of 6 mg/ml and stored at –20°C.

### Total muropeptides composition

The procedure used has been described elsewhere [30, 36]. The PG (200 μg) was digested with 20 μg of mutanolysin from *Streptomyces globisporus* (Sigma) for 18 h at 37°C in 12.5 mM sodium phosphate buffer (pH 5.8). The enzyme reaction was stopped by boiling the sample for 3 min, the digested PG was mixed with sodium borohydride (2 mg) for 15 min. The pH of the samples was then adjusted to 2.0, and the samples were centrifuged to remove insoluble material. We used a linear gradient from 50 mM sodium phosphate buffer (pH 4.3) to 50 mM sodium phosphate buffer (pH 5.1) containing 15% methanol for 120 min on a Hypersil ODS column (4.6 × 250 nm; 5 μm particles; ThermoHypersil-Keystone) at 52°C using a flow rate of 0.5 ml/min. UV detection was carried out at 205 nm. The quantity of each muropeptides was assessed by measuring the area of the corresponding peak. We only compared PGs that were extracted simultaneously. For assessing evolution of the PG structure in the different phyla, we determined for each species the total ratio of GM4/GM5 that is calculated as followed (GM4+GM4_GM4)/(GM5+GM5_GM4) where GM4_GM4 and GM5_GM4 represent dimers.

### Test of PG recognition by Nod proteins

Muropeptides recognition by Nod receptors has been performed as described [20, 21]. Briefly, in one well of 24 well plates, HEK293T cells grown to mid confluency were transfected using 1μl of Fugene with a mixture containing a NF-κB-luciferase reporter construct (50ng), a ß-galactosidase expressing vector (25ng) and the pCDNA3 plasmid (225ng), alone (None) or together with human Nod1 (hNod1) or human Nod2 (hNod2) or murine Nod1 (mNod1) (1ng each). Control muropeptides (MurTriDap as hNod1 agonist or MDP as hNod2 agonist or FK156 as mNod1 agonist, 100nM each) or PG to be tested (2, 1 or 0.5μg of non digested PG from the different bacteria) were added dropwise to the cells 30 min prior to the transfection in order to favor the intracellular delivery of the muropeptides and their subsequent recognition by the cytosolic Nod proteins, which results in activation of the NF-κB-driven luciferase expression. The luciferase and ß-galactosidase activities were measured 24h post stimulation/transfection from cellular lysates in the presence of their substrates. Data (mean values of triplicates) are expressed as relative light units, normalized to the β-galactosidase activity and are representative of two independent experiments.

### Electron microscopy

For scanning electronic microscopy, the strains were grown on GCB agar plates during 6h and prefixed in 2.5% glutaraldehyde in 0.1 M cacodylate buffer for at least 1h and then rinsed in 0.2 M cacodylate buffer. After post-fixation in 1% osmium tetroxide (in 0.2 M cacodylate), bacteria were dehydrated with increasing ethanol concentrations. Specimens were critical point dried using carbon dioxide, coated with gold and examined with a JEOL JSM-6700F scanning electron microscope. The ratio surface/volume was calculated as followed: for coccus, the radius perpendicular to the septum (r) and the diameter (d) parallel to the septum were measured and a mean radius (mr) was calculated as followed: (2r+d)/4. Finally, the ratio surface/volume was calculated using the following formula: [4π(mr)^2^]/[4/3π(mr)^3^]. For bacillus, as they are composed of a cylinder with a height (h) and a length (l) fused to two half sphere (see above for calculation), the ratio surface/volume was calculated using this formula: [4π(mr)^2^ + πlh]/[4/3π(mr)^3^ + π(l/2)²h].

For immune-gold labeling of GM5, freshly extracted PG (12 ug) from *N*. *bacilliformis* was incubated with 0.25 μg/ml of vancomycin (Sigma) in a small volume (4 μL) over-night. The samples were subsequently diluted 1/50 and laid on the grids by ultracentrifugation in a Beckman Airfuge (120 000g for 5 min). The grids were washed three times with PBS and blocked by incubation in 1% ovalbumine PBS for 5 min. The grids were subsequently incubated for 60 min with the Anti-vancomycin (ab19968; Abcam) serum diluted 1/150 in PBS-1% ovalbumine. After three washes and one additional blocking step with 1% ovalbumine, the grids were incubated with an anti-IgG mouse antibody (1/20 in PBS-1% ovalbumine) coupled with 6 nm gold beads. Finally, after three washes, the grids were stained with 3% PTA (phosphotungstic acid).

### Genome sequencing

Genomic DNA was extracted using Genomic Tip 20/G kit (Qiagen) from an overnight culture grown on G2 plates. Whole-genome sequencing was performed either using PacBio RSII (*N*. *elongata* wild-type) or Illumina HiSeq 2000 sequencer (which generated 150-bp paired reads; *N*. *elongata* Δ*mreBCD*,*pbpX*,*rodA*). The sequencing was done by GATC Biotech using standard protocols as recommend by the manufacturer’s instructions. For *N*. *elongata* wild-type, the sequences (2 cells) were *de novo* assembled using SMRT analysis v2.1.1 using default settings, obtaining wherein 1 contig with ≈200× average genome coverage. The genome was annotated using the NCBI Prokaryotic Genomes Automatic Annotation Pipeline (PGAAP) (http://www.ncbi.nlm.nih.gov/genomes/static/Pipeline.html). This Whole Genome project has been deposited at DDBJ/EMBL/GenBank under the accession CP007726. For *N*. *elongata* Δ*mreBCD*,*pbpX*,*rodA* the reads were aligned to CP007726 and analysed with defaults settings with seqman NGEN v12 (DNASTAR).

### Phylogenetic analysis of *Neisseriaceae* and *Moraxellaceae*

*Neisseriaceae*: the core-genome consists in the set of genes present in all genomes and was defined as the intersection of the pairwise lists of orthologs. Orthologs were identified as bidirectional best hits, using end-gap free global alignments, between the proteome of *Eikenella corrodens* ATCC 23834 as a pivot and each of the other 27 proteomes (see Touchon, GBE, 14 for details). Hits with less than 40% similarity in amino acid sequence or more than 20% difference in protein length were discarded. Each of the 343 families of proteins of the core-genome was used to produce a multiple alignment with muscle v3.52 (default parameters) [37, 38]. Poorly aligned regions were removed with BMGE using the BLOSUM62 matrix [39]. Alignments were then concatenated, producing an alignment with 107286 columns and 47462 patterns. The phylogenetic tree was inferred using the maximum-likelihood method implemented in IQ-Tree 1.2.2 [40]. Initially, we set the program to test all the 144 combinations of substitution models available in the program. Both the AIC and the BIC criteria pointed the LG+I+G4+F model as the one most appropriate for the dataset. Hence, we built the maximum likelihood tree using IQtree with this model and default parameters. We produced 1000 rapid bootstraps to assess the robustness of the topology. The tree was rooted with the outgroup *Snodgrassella alvi* wkB2.

*Moraxellaceae*: As only few genomes are available, the schematic phylogeny is based on 16s sequencing and alignment with Mauve of sequences from isolates from our collection and *M*. *bovis*.

In addition to 16s sequencing, all the strains used in this study were verified for the presence of *rodA* in the case of *Neisseriaceae* and *pbpX* in the case of *Moraxellaceae* using the family specific primers (RodAF-RodAR and PbpXMcF-PbpXMcR respectively).

### Genome comparison

To detect genes deletion or insertion in *Neisseriaceae* concomitant with cell shape change, it was necessary to determine genes that do or do not have orthologues among all the available genomes. We used complete genomes of *N*. *meningitidis* MC58, *N*. *gonorrhoeae* 8013, *N*. *lactamica* 020–06, *N*. *elongata* ATCC 29315 and incomplete genomes of *N*. *lactamica* ATCC 23970, *N*. *polysaccharea* ATCC 43768, *N*. *cinerea* ATCC 14685, *N*. *subflava* NJ9703, *N*. *mucosa* C102, *N*. *flavescens* SK114, *N*. *flavescens* NRL300031/H210, *N*. *sp* GT4A CT1, *N*. *sicca* VK64, *N*. *macacae* ATCC 33926, *N*. *sicca* ATCC 29256, *N*. *mucosa* ATCC 25996, *N*. *sp*. oral taxon 014, *N*. *bacilliformis* ATCC BA1200, *N*. *weaveri ATCC 51223*, *N*. *weaveri* LMG5135, *Simonsiella muelleri* ATCC 29453, *Kingella oralis* ATCC 51147, *K*. *kingae* ATCC 23330, *K*. *denitrificans* ATCC 33394, *Snodgrassella alvi* wkB2, *N*. *shayeganii* 871, and *Eikenella corrodens* ATCC 23834. We excluded *Neisseria wadsworthii* 9715. We performed an alignment search with the StandAlone TBLASTN program [41], using the 2063 predicted proteins from *N*. *meningitidis* MC58 or 2105 predicted proteins from *N*. *elongata* ATCC29315 as the query sequences to search for matches in the genomic DNA of other organisms. We obtained two matrix of around 40000 scores (2063 or 2105 protein sequences blasted against 18 genomes) providing two types of output: categorical (hit versus no hit) and quantitative (degree of similarity). To categorically assign that there was no hit, we employed the default E-value (or Expectation value) of e^-10^ which is provided at NCBI and has been used in a similar study[42]. Thus, if the statistical significance ascribed to a comparison is greater than this E value, we assigned a percentage of similarity of 0% to that comparison.

To analyze quantitative results, we used MycoHIT [16] to assign absence or presence of an orthologue as previously described. “Absence” was defined as lower values and “presence” as higher values, than the 95 percentile of tested species.

The presence or absence in other bacteria of orthologs of YacF, MreB, MreD and RodZ, were identified using String database [43] based on the Clusters of Orthologous Groups (COG) method [44] as previously described [45].

### RNA sequencing and qRT-PCR validation

RNA was extracted using QIAGEN RNeasy mini Kits. Enriched mRNA was obtained from 7 μg of total RNA using the rRNA capture hybridization approach from the MicrobExpress kit (Ambion), according to the manufacturer's instructions. For strand-specific high-throughput sequencing, directional cDNA libraries were prepared from enriched fragmented mRNA using the TruSeq Stranded mRNA LT sample preparation kit, set A (Illumina). Fragments of cDNA of ± 150 bp, ligated with Illumina adapters and amplified per PCR, were purified from each library. Quality was confirmed on a Bioanalyzer (Agilent) and quantification done using a Qubit dsDNA HS Assay Kit (Invitrogen). Sequencing of 51 bases was performed in single-end mode, using an Illumina HiSeq2000 instrument (Illumina). Reads were cleaned from the adapter sequences and from sequences of low quality using an in-house program. Only sequences with a minimum length of 25 nucleotides were considered for further analysis. The Seqman NGEN (DNASTAR) was used to align the reads to the *N*. *elongata* CP007726 genome.

qRT-PCR was performed using Power SYBR Green PCR master mix and StrepOne plus (Applied Biosystems) using the primers listed in S1 Table. A ΔΔCt was calculated by subtracting with ΔCt of *gyrA*. In addition, a standard t-test was applied to assess statistically significant difference in comparison to the wild-type ΔΔCt.

### Statistics

A t-test was used to determine statistical significance of observed differences (GraphPad Prism v5.0; GraphPad Software, CA). For RNAseq, count data were analyzed using DNASTAR Qseq program. Data were normalized using RPKM method (Summarize and normalize all RNA-Seq experiments by assigned Reads Per Kilobase of template per Million mapped reads). The generalized linear model was set with condition "WT" as the reference. Raw p-values were adjusted according to the Benjamini and Hochsberg (BH) procedure and genes with an adjusted p-value below 0.001 and a 3 fold differences were considered differentially expressed.

## Supporting Information

S1 Fig qRT-PCR showing lack of *yacG* miss-regulation in *N*. *elongata* ∆*yacF*. Graphical representation of ΔΔCt calculated by subtracting the ΔCt of indicated genes with the ΔCt of *gyrA*.
(PDF)

S2 Fig Growth curves of *N*. *elongata* wild-type and mutants. Dry weight measured at different times for culture of *N*. *elongata* wild type and mutants grown in liquid media at 37°C with agitation.
(PDF)

S3 Fig Muropeptides composition quantification of *N*. *elongata* wild-type and mutants. rp-HPLC analyses of muropeptides generated by mutanolysin digestion of PG from *N*. *elongata* and mutants. Each percentage corresponds to the peak UV area over the total muropeptide UV peak area. The name of the muropeptide is indicated if known. Otherwise, elution time is indicated.
(PDF)

S1 Table Primers used in this study. (DOCX)

S2 Table List of genes detected as potentially inserted or deleted at node 2. The percentage represents the similarity score of the query protein sequences (*N*. *elongata* or *N*. *meningitidis*) matched with the translated genomic DNA of other organisms. The product name and the length of the proteins are also indicated.
(XLS)

S3 Table Results of transcriptomic accessed by RNA sequencing. List of genes found up or down regulated in the different mutants compared to wild type *N*. *elongata*.
(XLS)
