# Supplementary figures and images for: Common Cell Shape Evolution of Two Nasopharyngeal Pathogens
Source: PLoS Genet. 2015 Jul 10;11(7):e1005338. doi: 10.1371/journal.pgen.1005338 (PMC4498754; doi:10.1371/journal.pgen.1005338)

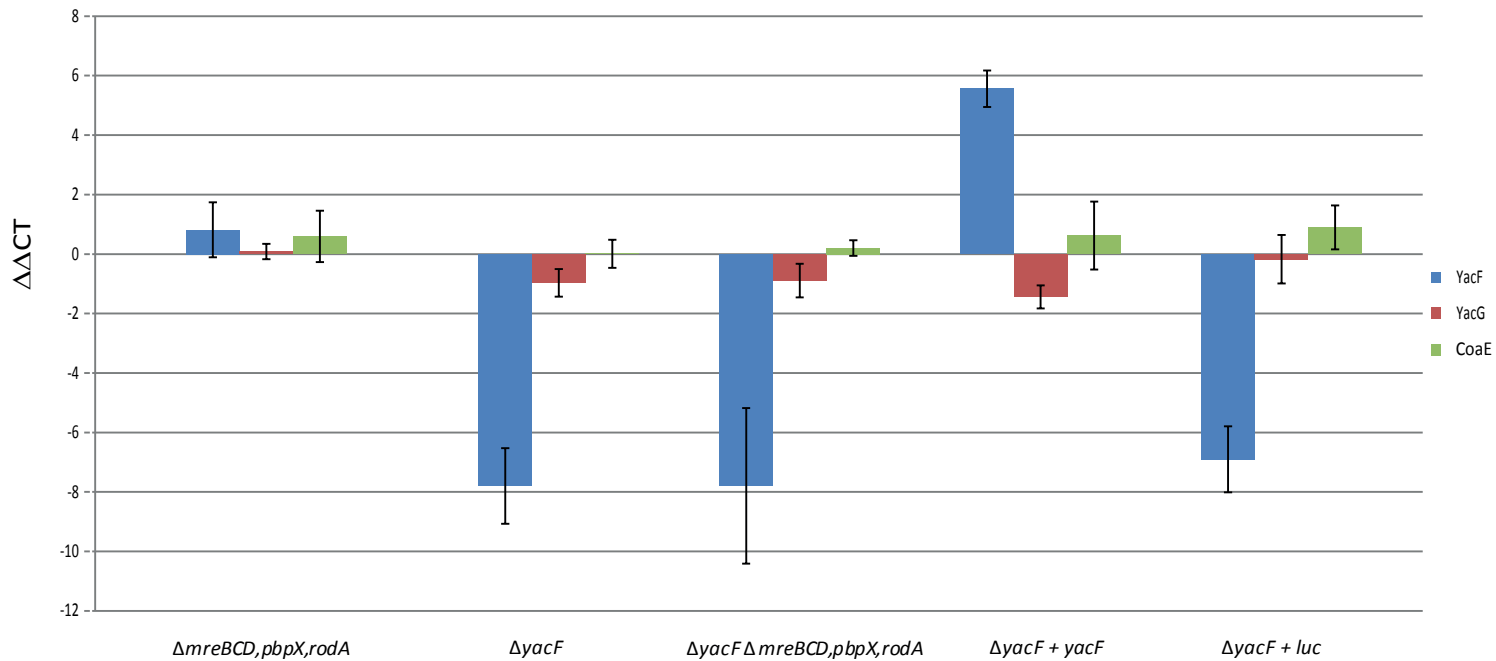

Supplement: S1 Fig — Graphical representation of ΔΔCt calculated by subtracting the ΔCt of indicated genes with the ΔCt of gyrA. (PDF) [file pgen.1005338.s001.pdf]

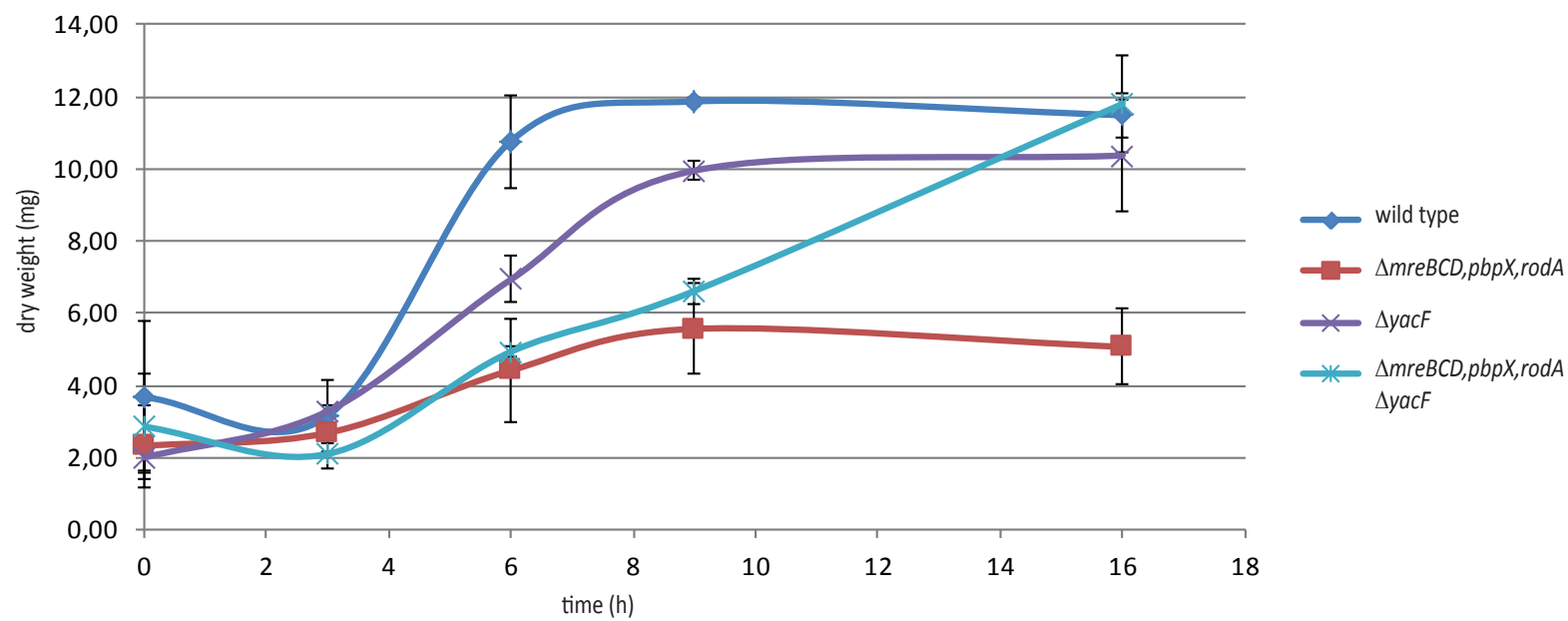

Supplement: S2 Fig — Dry weight measured at different times for culture of N. elongata wild type and mutants grown in liquid media at 37°C with agitation. (PDF) [file pgen.1005338.s002.pdf]
