# Supplementary material for: Common Cell Shape Evolution of Two Nasopharyngeal Pathogens
Source: PLoS Genet. 2015 Jul 10;11(7):e1005338. doi: 10.1371/journal.pgen.1005338 (PMC4498754; doi:10.1371/journal.pgen.1005338)
Supplement: S1 Table — (DOCX) [file pgen.1005338.s004.docx]

**Table S1.** Oligonucleotide primers used in this study

| Primers name | Sequence (5’→ 3’) |
| --- | --- |
| 5’RD1NeF | tttcccgggacggcacacggtttcc |
| 5’RD1NeR | CATTCTAGAAATAACGTGTGATGAAACGC |
| 3’RD1NeF | GCGGGATCCTGCCCGTGGTCGGC |
| 3’RD1NeR | CAAACTAGTCTTGGCGCAGTTTGCCC |
| 5’RD2NeF | TTGCTGCAGTCTGTTATGGATGGTCTTG |
| 5’RD2NeR | AGTATGCATCTCAATATCCGTTTCTAACC |
| YacGF | GGAacgcgtGAGGCCGATATGCGAAAAG |
| YacGR | GCAACTAGTTCAAACGTAGCTAATTTTTTG |
| KmXbaIR | ATTCAGTCTAGAGCCGTCTGAATGCTTTTTAGACATCTAAAT |
| 3’RD2NeMoinsF | ATTCCATGGGGTTGGTAAATAAAATTCGG |
| 3’RD2NeR | AACGCATGCTGCTGTATCGGAAAATTC |
| 5nrqF | GGGACTAGTGTCGTCCTACATCTGGTCGC |
| 5nrqR | AAGGGATCCGCTAGCTCCAAAAAAGCCATCACAAGGCTGTCC |
| 3nrqF | GTATCTAGAAGAAAAAGGCATCATTATGCTG |
| 3nrqR | CGTCCATGGGGATGTTTTCGTCTTCCACGC |
| pilEpF | CTATGCTAGCGCGCAAATTCAAAAATCTCAAATTCCGAC |
| pilEpR | GTGTCCATGGAATTACTCCTAATTGGAAAGGAAATGC |
| yacFNcoIF | TTGCCATGGTTACTTTCGAACACCCTTTG |
| yacFPstIR | ATGCTGCAGCGCGGACATTTTACTTTTC |
| 5’KORD2NbF | GGGCTGCAGGCCAACTACGGCGGC |
| 5’KORD2NbR | TGTATGCATGGTGTCAGGGTGTGGGATG |
| 3’KORD2NbF | TCGCCATGGCCGAAGAACGCCCGC |
| 3’KORD2NbR | GCGGCATGCGGTGTCGGGTCGGC |
| RodAF | CAATCATGGATTGGATGATGTGGTAGCC |
| RodAR | ATCCAACCTTCCGAAATCATGAAAATCG |
| PbpXMcF | TCTCACGTTGGAGAATCCAGTAATCAAA |
| PbpXMcR | ATCCATCGCCCAAGGCAAAAGCTATAA |
| RTyacFF | attttgcaggaattggaacg |
| RTyacFF | taatcgccatcaaccattca |
| RTyacGF | aggccgatatgcgaaaagta |
| RTyacGR | aaaatcttcctgatcaatcatcg |
| RTcoaEF | ggtttttgcagacagcagtg |
| RTcoaER | agccatccaataccttgagc |
| RTrodZF | aaaatgccaagcaaacttcg |
| RTrodZR | cttgggacaaagcctgtgat |
| RTPbp3F | AGCTTCATCAACCAATGGCG |
| RTPbp3R | GTTTGTTCATGTCGGCCAGT |
| RTrodAF | ACGCCACCSGARCCRATGGCAATCAT |
| RTrodAR | GCCTCTGATGTGGAAYTAYGGSATGCA |
| RTpglDF | TGATTTTCTGCGCCTTCTGG |
| RTpglDR | GCACAAACAGCAAGACCGTA |
| RTftsAF | GGCCGAATTGATGGCTGATT |
| RTftsAR | CGATATCCGCCTGACTGACT |
| RTftsQF | AAATCCGATTGAGTGAGCGC |
| RTftsQR | TGTCCTTTGAATTGCGGCAA |
| RTalrA2 | gggtgaagtttccgcctttt |
| RTalrA2 | ttcgacctctaccgcttctc |
| RTamiCF | ATGTCGCCTTTGTGCAACTT |
| RTamiCR | AAAACCGGCGGAAACAAAGA |
| RTmurIF | GCCGGTTTTGGACGTAATCA |
| RTmuriR | TGCTGTTTACCGTGGTGTTG |
| RTgyrAF | gcaaccatctacggcttgag |
| RTgyrAR | atgatgatggcttcgcgttc |
