# Supplementary material for: Common Cell Shape Evolution of Two Nasopharyngeal Pathogens
Source: PLoS Genet. 2015 Jul 10;11(7):e1005338. doi: 10.1371/journal.pgen.1005338 (PMC4498754; doi:10.1371/journal.pgen.1005338)
Supplement: S3 Fig — rp-HPLC analyses of muropeptides generated by mutanolysin digestion of PG from N. elongata and mutants. Each percentage corresponds to the peak UV area over the total muropeptide UV peak area. The name of the muropeptide is indicated if known. Otherwise, elution time is indicated. (PDF) [file pgen.1005338.s003.pdf]

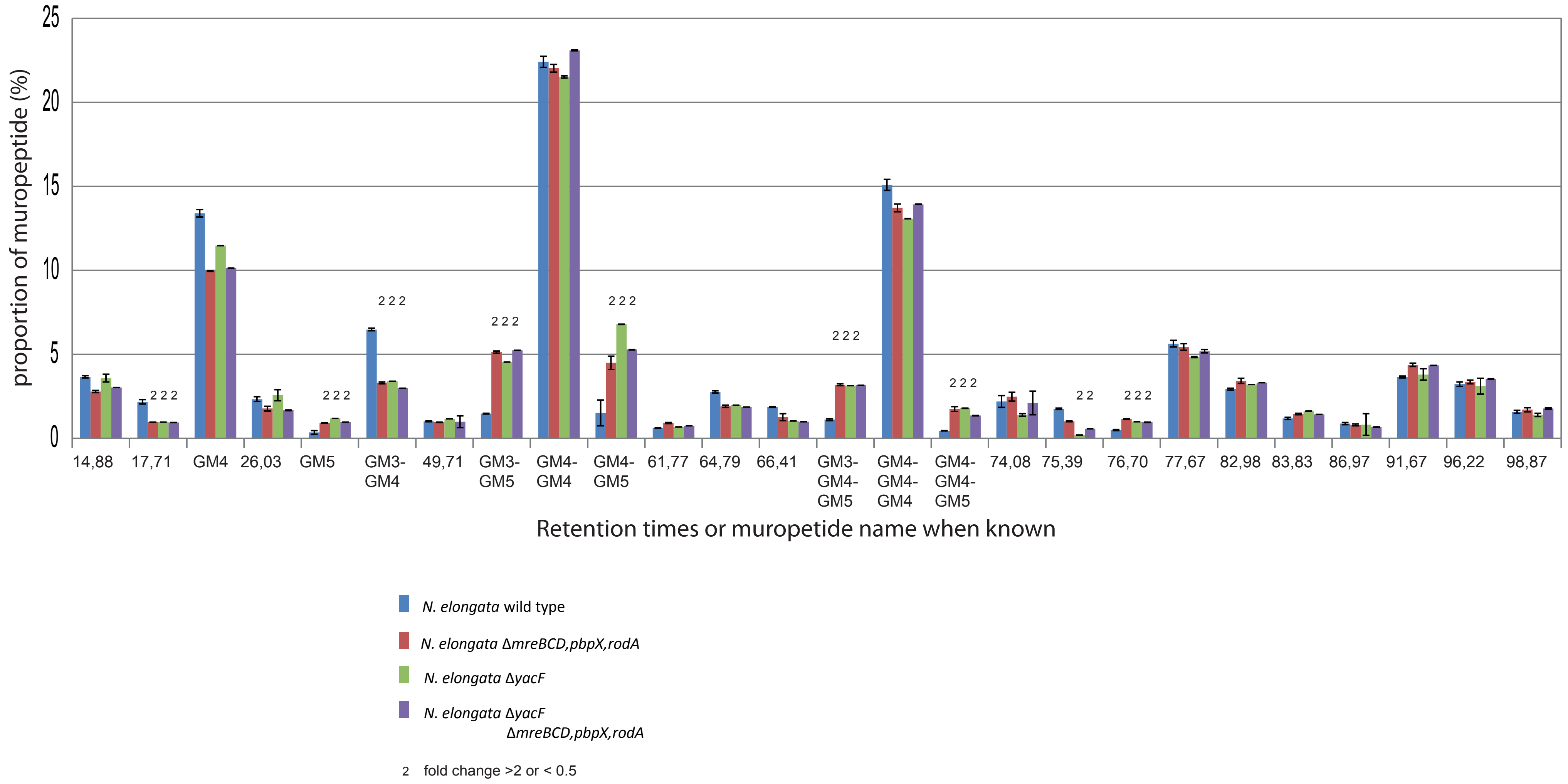
proportion of muropeptide (%)
25
20
15
10
5
0
2 2 2
14,88
17,71
GM4
26,03
GM5
GM3-GM4
49,71
GM3-GM5
GM4-GM4
GM4-GM5
61,77
64,79
66,41
GM3-GM4-GM5
GM4-GM4-GM4
GM4-GM4-GM5
74,08
75,39
76,70
77,67
82,98
83,83
86,97
91,67
96,22
98,87
Retention times or muropetide name when known
N. elongata wild type
N. elongata ΔmreBCD,pbpX,rodA
N. elongata ΔyacF
N. elongata ΔyacF ΔmreBCD,pbpX,rodA
2 fold change >2 or < 0.5
